# Lignans Isolated From Flower Buds of *Magnolia fargesii* Attenuate Airway Inflammation Induced by Cigarette Smoke *in vitro* and *in vivo*

**DOI:** 10.3389/fphar.2018.00970

**Published:** 2018-09-07

**Authors:** Su-Ui Lee, Hyung Won Ryu, Seoghyun Lee, In-Sik Shin, Ji-Hee Choi, Jae-Won Lee, Jinhyuk Lee, Mun Ock Kim, Hyun-Jun Lee, Kyung-Seop Ahn, Sung-Tae Hong, Sei-Ryang Oh

**Affiliations:** ^1^Natural Medicine Research Center, Korea Research Institute of Bioscience and Biotechnology, Cheongju, South Korea; ^2^College of Bioscience and Biotechnology, Chungnam National University, Daejeon, South Korea; ^3^College of Veterinary Medicine (BK21 Plus Project Team), Chonnam National University, Gwangju, South Korea; ^4^Genome Editing Research Center, Korea Research Institute of Bioscience and Biotechnology, Daejeon, South Korea; ^5^Department of Anatomy and Cell Biology, Department of Medical Science, College of Medicine, Chungnam National University, Daejeon, South Korea

**Keywords:** CS, COPD, EGFR, ERK, Akt, *Magnolia fargesii*, lignans

## Abstract

The flower buds of *Magnolia fargesii*, known traditionally as Xinyi, exert anti-inflammatory effects against inflammatory lung diseases such as COPD. Lignans isolated from Xinyi are an important group of plant-derived anti-inflammatory compounds. However, the mechanisms of action underlying their protective effects against COPD are not yet fully understood. Here, we showed that seven lignans (lignans 1–7) obtained from a CHCl_3_ fraction of Xinyi effectively suppress the inflammatory response in CSC-stimulated airway epithelial cells (*in vitro*) and in a mouse model of COPD established by exposure to CS and LPS. The CHCl_3_ fraction was found to inhibit CSC-induced IL-6 expression in human airway epithelial cells and to suppress the infiltration of inflammatory cells (neutrophils and macrophages) and secretion of inflammatory cytokines, such as tumor necrosis factor-α (TNF-α) and interleukin-6 (IL-6) in the mouse model. Similarly, each of the seven lignans isolated from the CHCl_3_ fraction also suppressed the infiltration of inflammatory cells (neutrophils and macrophages) and secretion of inflammatory mediators such as reactive oxygen species (ROS), TNF-α, and IL-6 *in vivo*. Notably, all lignan compounds significantly suppressed both extracellular signal-related kinase (ERK) and Akt phosphorylation levels in CSC-stimulated human lung mucoepidermoid carcinoma (NCI-H292) cells. Of these, lignan 1 (dimethylpinoresinol) inhibited the expression of CSC-induced inflammatory cytokines (IL-1β, -6, and -8) *in vitro* in a dose-dependent manner by suppressing the activation of epidermal growth factor receptor (EGFR) and its downstream effectors, including ERK and Akt, in NCI-H292 cells. Our results show that the lignans isolated from Xinyi may prevent airway inflammatory diseases through the suppression of EGFR and its downstream effectors.

## Introduction

COPD is a progressive lung disease characterized by airway inflammation, mucus hypersecretion, and a persistent cough (Angelis et al., 2014). It is predicted to become the third-leading contributor to global disease burden by the year 2030 (Lin et al., 2015). Although good outcomes have been achieved in COPD patients using combination treatment involving ICSs and diverse bronchodilators, ICS use leads to adverse effects such as pneumonia and osteoporosis. Thus, more effective and safer drugs need to be developed to manage COPD (Hanania et al., 1995). Recently, medicinal plants that show anti-inflammatory activity have garnered interest in the treatment of COPD (Sharafkhaneh et al., 2007). In fact, some of these herbal remedies have been demonstrated to be effective and safe alternatives (or complements) to standard therapies for lung inflammatory disease (Li, 2007; Zhao et al., 2015).

The *Magnolia* genus of herbs has been traditionally used to treat inflammatory diseases such as headache, sinusitis, and allergic rhinitis (Gao et al., 2012). In particular, the dried flower buds of *M. fargesii*, or Xinyi, exhibit efficacy in treating airway inflammatory diseases including asthma, bronchitis, and emphysema (Park et al., 2012; Oh et al., 2016). Xinyi contains several components with promising biological activity, such as essential oils (Fang et al., 2004), lignans (Lee et al., 2007), neolignans (Schuhly et al., 2009), and sesquiterpenes (Jung et al., 1998). Of these, lignans are an important class of naturally occurring plant compounds exhibiting potent anti-inflammatory properties (Bastos et al., 2001). However, the mechanisms of action underlying their protective effects against COPD are not yet fully understood.

The levels of inflammatory mediators and the numbers of inflammatory cells found in the BALF isolated from cigarette smokers are high (O’Donnell et al., 2006). Since CS induces an elevated release of inflammatory mediators such as cytokines [interleukin-6 (IL-6) and tumor necrosis factor-α (TNF-α)] and reactive oxygen species (ROS) in the lung, CS can be considered the primary risk factor for COPD (Barnes, 2004). In mice, inhalation of both CS and LPS are a combination (CS/LPS) that accelerates inflammatory responses, resulting in lung injury similar to that seen in COPD patients (O’Donnell et al., 2006; Moazed et al., 2016). These observations led to the establishment of an *in vivo* COPD-like mouse model, in which animals are exposed to CS and LPS. This *in vivo* COPD-like mouse model can be employed in the preclinical investigation of therapeutic strategies to identify effective ways to inhibit CS-induced inflammatory lung disease in COPD patients (Durham and Adcock, 2015).

Generally, CSC, which contains the majority of harmful tobacco constituents, induces activation of mitogen-activated protein kinases (MAPKs) and phosphoinositide 3-kinase (PI3K)/Akt pathway (Yang et al., 2009), which are the major signaling components involved in COPD pathogenesis (Geraghty et al., 2014). The group of MAPK molecules contains three principal serine/threonine protein kinases – extracellular receptor kinase-1/2 (ERK1/2), c-Jun N-terminal kinases (JNK), and p38 MAPK – that are the key regulators of inflammatory responses (Thalhamer et al., 2008). Of these, ERK1/2 kinases trigger the release of inflammatory mediators such as IL-6, TNF-α, and ROS in airway epithelial cells (Hellermann et al., 2002; Mercer and D’Armiento, 2006). Recent studies have shown that magnolin, one of the lignan compounds isolated from Xinyi, exhibits anti-cancer activity through inhibition of ERK1/2 in lung epithelial cells (Lee et al., 2014, 2015b). The PI3K/Akt pathway also plays an important role in lung inflammation (Medina-Tato et al., 2007). Total PI3K activity is determined by the phosphorylation level of its downstream target Akt, which is involved in the regulation of cell proliferation, cell transformation, and cancer development. Total PI3K activity is markedly increased in peripheral lung tissue and in macrophages from patients with COPD (To et al., 2010). Indeed, inhibitors of PI3K (for example, aerosolized TG100-115) repressed the inflammatory responses in CS-exposed mice (Doukas et al., 2009). In addition, aschantin, another lignan compound from Xinyi, inhibits the activation of Akt (Lee et al., 2014, 2015b). Therefore, Akt and/or ERK signaling cascades may be good targets for anti-inflammatory therapeutic modalities that may be used in the treatment of inflammatory lung diseases such as COPD (Vallath et al., 2014).

The epidermal growth factor receptor (EGFR) is a member of the erythroblastic oncogene B (ErbB)/HER family of receptors regulating lung homeostasis and respiratory diseases. Deregulation of EGFR signaling is related to airway inflammatory diseases such as asthma, COPD, and cystic fibrosis (Vallath et al., 2014). Since CS can induce ligand-independent phosphorylation of EGFR through the activation of c-Src, a non-receptor tyrosine kinase, which subsequently activates its own downstream effectors, such as MEK/ERK (Mercer and D’Armiento, 2006) and PI3K/Akt (Khan et al., 2008; Yang et al., 2009; Geraghty et al., 2014), regulation of the EGFR signaling cascade may be a promising therapeutic approach in the treatment of respiratory lung diseases (Vallath et al., 2014).

In this study, we isolated seven lignan compounds from a CHCl_3_ fraction of Xinyi and demonstrated that they exert effective anti-inflammatory activity in both CSC-stimulated human airway epithelial cells and in a mouse model of CS/LPS-induced COPD. These seven Xinyi lignans exhibit anti-COPD activity through the inhibition of both ERK and Akt signaling pathways. Moreover, lignan 1 (dimethylpinoresinol) exhibited anti-inflammatory activity through the suppression of CSC-activated EGFR and its downstream effectors, including ERK and Akt, in human airway epithelial cells.

We propose that the lignans isolated from Xinyi are potential therapeutic agents for treating inflammatory lung diseases such as COPD.

## Materials and Methods

### Instruments and Reagents Used

Optical rotation was measured using a Jasco P-1020 polarimeter (Jasco, Tokyo, Japan). Nuclear magnetic resonance (NMR) spectra were recorded on a Bruker (AM 500 MHz) FT-NMR spectrometer using tetramethylsilane as an internal standard. High-resolution electrospray ionization mass spectrometry (HRESIMS) was performed using a Waters Q-TOF Premier spectrometer. All solvents used for column chromatography were of analytical grade (SK Chemicals Co., Ltd., Seongnam-si, Korea). The solvents used for ultra-performance liquid chromatography (UPLC) were of liquid chromatography/mass spectrometry (LC/MS) grade (SK Chemicals Co., Ltd.).

### Plant Material and Active Fraction Preparation

Flower buds of *Magnolia fargesii* (Xinyi), collected in China, were provided by Jinheung Herb Factory^1^ in August 2014. Xinyi (8.0 kg) were extracted with methanol at room temperature three times to obtain approximately 1.2 kg of solid extract. This MeOH extract was suspended in water and partitioned using solvents of increasing polarity to generate *n*-hexane, CHCl_3_, *n*-butanol, and H_2_O-soluble extracts, respectively. The CHCl_3_ fraction was selected as an active fraction and used in the next isolation step. NMR, MS, and UPLC chromatograms for the isolated lignans from the CHCl_3_ fraction are described in the **Supplementary Material** (methods illustrated in **Supplementary Figure S2**).

### Chemicals and Reagents

Cigarette smoke condensate was purchased from the Tobacco and Health Research Institute (University of Kentucky, Lexington, KY, United States). Anti-ERK and anti-phospho-ERK antibodies were purchased from Santa Cruz Biotechnology (Santa Cruz, CA, United States). Anti-Akt and anti-phospho-Akt antibodies were obtained from Cell Signaling Technology (Beverly, MA, United States). Roflumilast, U0126, Wortmannin, and LPS (from *E. coli* serotype 0111:B4) were purchased from Sigma (St. Louis, MO, United States).

### Cell Preparation and Culture

NCI-H292 cells, a human pulmonary muco-epidermoid carcinoma line, were acquired from the American Type Culture Collection (CRL-1848; ATCC, Manassas, VA, United States). Early passages (passage number 7–20) were used for all experiments. Cells were cultured in Roswell Park Memorial Institute-1640 medium (RPMI-1640; Hyclone, GE Healthcare, United Kingdom) supplemented with 10% fetal bovine serum (FBS; Hyclone) and 100 units/mL penicillin plus 100 μg/mL streptomycin (Hyclone) at 37°C under a humidified 5% CO_2_ atmosphere. For enzyme linked immunosorbent assay (ELISA) of IL-6 production, NCI-H292 cells were seeded in 24-well plates at a density of 1 × 10^5^ cells for 16 h. They were then transferred to reduced-serum medium (0.1% FBS). After a 16 h incubation period, the cells were treated with different fractions from Xinyi or the seven purified lignans from the CHCl_3_ extract for 2 h before adding CSC (20 μg/mL). After addition of CSC, the cells were further incubated for 12 h. The presence of cytokines in the supernatant was analyzed using IL-6, IL-1β, and IL-8 ELISA kits according to the manufacturer’s instructions (R&D, Minneapolis, MN, United States).

### Cell Viability Assay

NCI-H292 cells were seeded and cultured in 96-well plates in RPMI-1640 medium at a density of 5 × 10^3^ cells/well for 16 h. The medium was subsequently replaced with reduced-serum medium (0.1% FBS). After a 16-h incubation, the cells were cultured with corresponding concentrations of different fractions or seven lignans for 24 h. In addition, to measure cell viability in a condition of high cell proliferation, cells were cultured in 96-well plates in RPMI-1640 medium at a density of 5 × 10^3^ cells/well. After a 16 h incubation, the cells were cultured with different concentrations of seven lignans with fresh medium (10% FBS) for 24 h. Cell growth was measured in triplicate using a Cell Counting Kit-8 (Dojindo Molecular Technologies, Rockville, ML, United States) according to the manufacturer’s protocol. Optical absorbance was determined by a VersaMax Microplate Reader (Molecular Devices, Sunnyvale, CA, United States) and used to calculate percentage (%) of the control value for each condition.

### Western Blot Analysis and ELISA

NCI-H292 cells (5 × 10^5^ cells/well) were cultured in six-well plates. The cells were incubated for 16 h in GM, which was subsequently replaced by serum-free medium. After 16 h of incubation, the cells were pretreated with the indicated concentrations of compounds for 2 h and subsequently treated with CSC (50 μg/mL) for 30 min. At least 30 μg of the whole cell lysate protein was prepared and loaded as described previously (Lee et al., 2016). Protein bands were visualized using a LAS-4000 luminescent image analyzer (Fujifilm, Tokyo, Japan) and quantified by densitometry (Fuji Multi Gauge software version 3.0). The lung tissues were homogenized in a RIPA lysis buffer (1/10 w/v) containing a protease inhibitor cocktail (Sigma, St. Louis, MO, United States) (Lee et al., 2018). For ELISA, the activities of phospho-ERK1/2 were assayed using commercially available ELISA kits (Abcam, Cambridge, United Kingdom) according to the manufacturer’s instructions.

### *In vivo* Model for CS and LPS-Induced Airway Inflammation

Specific pathogen-free male C57BL/6 (6 weeks old; Koatech Co., Pyeongtaek, Korea) mice underwent whole-body exposure to fresh air or to the CS of eight cigarettes (3R4F research cigarettes; Tobacco and Health Research Institute, University of Kentucky, Lexington, KY, United States) for 1 h per day for 7 days using a CS generator (Daehan Biolink, Inchun, Korea). LPS (5 μg dissolved in 50 μL distilled water) was intra-nasally administered on Day 4. Roflumilast (a phosphodiesterase 4 inhibitor) is an approved medicine for the treatment of COPD (Pinner et al., 2012) and used as a positive control agent in this study. All drugs were administered to animals for 7 days by oral gavage 1 h before CS exposure. We performed two-animal experiments to evaluate the anti-inflammatory effects of the lignans extracted from Xinyi. For the first experiment, mice were randomly divided into five groups (*n* = 6 per group): NC (normal control), COPD (CS and LPS exposure), roflumilast (CS and LPS exposure + 10 mg/kg of roflumilast), CHCl_3_ 15, and CHCl_3_ 30 (CS and LPS exposure + 15 and 30 mg/kg of the CHCl_3_ fraction, respectively). For the second experiment, mice were divided into 10 groups (*n* = 6 per group): NC (normal control), COPD (CS and LPS exposure), roflumilast (CS and LPS exposure + 10 mg/kg of roflumilast), and lignans 1 to 7 (CS and LPS exposure + 15 mg/kg of each of the seven lignans).

### Analysis of BALF

After a 48-h period following LPS intranasal instillation, the animals were sacrificed by intraperitoneal injection of pentobarbital (50 mg/kg; Hanlim Pharm, Co., Seoul, Korea). A tracheostomy and BALF sampling were performed as previously described (Song et al., 2015). To measure the number of inflammatory cells in BALF samples, we collected all of inflammatory cells from the total volume of BALF fluid using Cytospin (Thermo Fisher Scientific). The cells were stained on a slide using Diff-Quik^®^ reagent (SYSMEX, Kobe, Japan). Inflammatory cell counts were determined by averaging cell numbers counted on five different places on a slide using a light microscope with a magnification of ×400 (Shin et al., 2015; Park et al., 2016, 2017). The ROS levels were determined using DCF-DA (Sigma-Aldrich, Carlsbad, CA, United States) based on a protocol from a previous report (Song et al., 2015). In addition, the levels of IL-6 and TNF-α in the BALF were quantified by ELISA, which was performed according to the manufacturer’s protocols (BD Biosciences, San Jose, CA, United States).

### Ethics Statement

All of the animal experiments were approved by the Institutional Animal Care and Use Committee (IACUC) of the Korea Research Institute of Bioscience and Biotechnology (KRIBB-AEC-17094). We performed all animal work in compliance with NIH Guidelines for the care and use of laboratory animals and with Korean national laws regarding animal welfare.

### Statistical Analysis

Data are presented as means ± standard deviation (SD). Statistical significance was analyzed by two-tailed Student’s *t*-test for *in vitro* experimental results. Significance was assumed when ^∗^*p* < 0.05, ^∗∗^*p* < 0.01, and ^∗∗∗^*p* < 0.001. One-way ANOVA followed by Dunnett’s multiple comparison test were used for analysis of the results from *in vivo* experiments. Single (^∗^), double (^∗∗^), and triplet (^∗∗∗^) asterisks represent statistical significance *p* < 0.05, *p* < 0.01, and *p* < 0.001, respectively.

## Results

### Anti-inflammatory Effects of the Active Fraction Extracted From Xinyi

**FIGURE 1:**
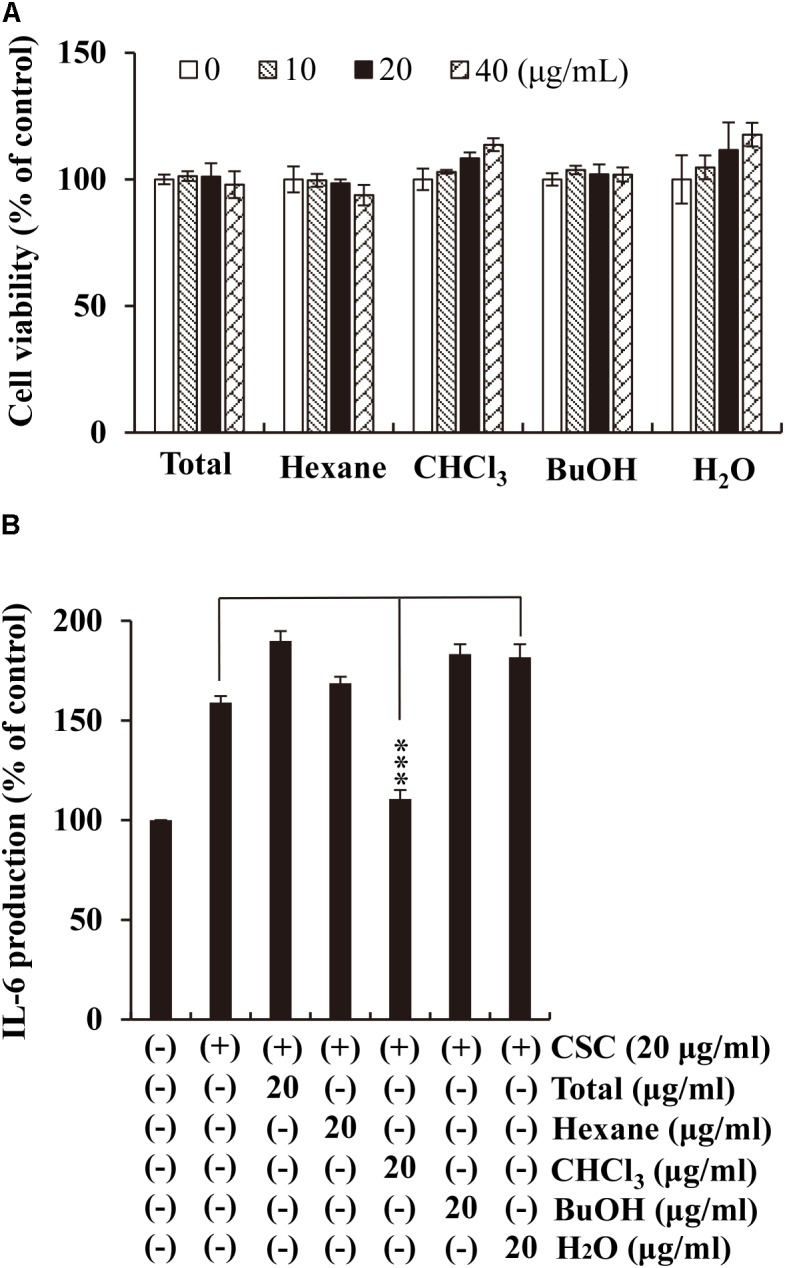
Effects of the CHCl_3_ fractions extracted from Xinyi on CSC-induced IL-6 production in human airway epithelial (NCI-H292) cells. **(A)** No adverse effects were caused by the different fractions from Xinyi on NCI-H292 cell viability. **(B)** The suppressive effect of the CHCl_3_ fraction on IL-6 expression was assayed by ELISA. Cells were pretreated with the different fractions (20 μg/mL) for 2 h and subsequently exposed to CSC (20 μg/mL) for 12 h. Bar graphs represent means ± SD of three independent experiments (^∗∗∗^*p* < 0.001 for comparison with controls, which were treated with CSC alone). Total, methanolic extract; Hexane, *n*-hexane fraction of the total extract; CHCl_3_, chloroform fraction of the total extract; BuOH, *n*-butanol fraction of the total extract; H_2_O, aqueous residue after partition of the total extract.

Exposure to CS, recognized as the most prominent risk factor for COPD, increases IL-6 expression level in the sputum of COPD patients (Wedzicha et al., 2000). To verify whether the different fractions (*n*-hexane, CHCl_3_, *n*-butanol, and water) extracted from Xinyi have anti-inflammatory activity that counters the effects of CS exposure, we measured IL-6 levels in CSC-stimulated NCI-H292 cells using ELISA. Prior to this, we evaluated the cytotoxicity of the fractions in NCI-H292 cells. The cells were cultured with increasing concentrations (10, 20, and 40 μg/mL) of the Xinyi fractions for 24 h. The fractions were found to show no cytotoxicity at any of the concentrations (**Figure 1A**). We employed a concentration of 20 μg/mL in subsequent experiments. As shown in **Figure 1B**, CSC significantly enhanced IL-6 secretion in NCI-H292 cells. This increase in IL-6 levels was most prominently suppressed by the CHCl_3_ fraction. This result indicates that the CHCl_3_ fraction extracted from Xinyi is its active fraction, containing the biologically active compounds with anti-inflammatory activity against CS.

### Anti-inflammatory Effects of the Active CHCl_3_ Fraction Extracted From Xinyi in CS- and LPS-Exposed Mice

**FIGURE 2:**
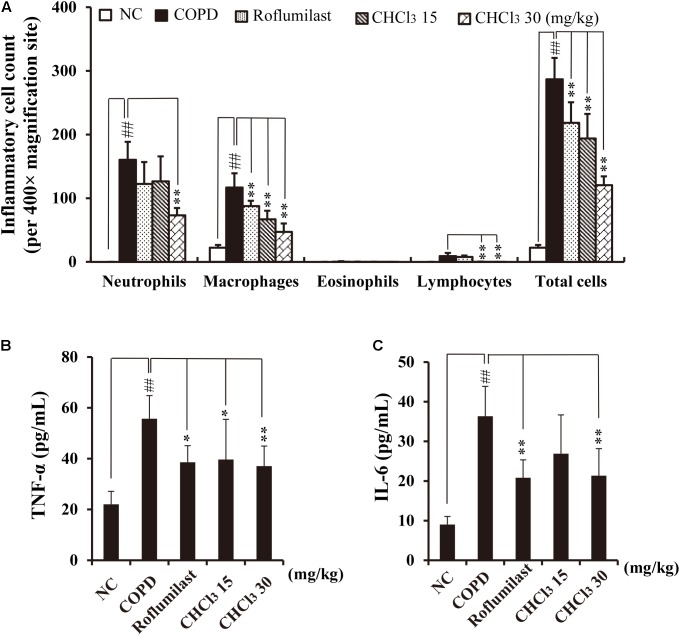
Effects of the CHCl_3_ fraction extracted from Xinyi on airway inflammatory responses in an *in vivo* COPD mouse model. **(A)** Inflammatory cell counts in BALF, **(B)** TNF-α production in BALF, **(C)** IL-6 production in BALF. NC, normal control mice; COPD, CS and LPS (CS/LPS)-exposed mice; Roflumilast, CS and LPS-exposed mice treated with roflumilast (10 mg/kg); CHCl_3_ 15 and CHCl_3_ 30, CS/LPS-exposed mice treated with the CHCl_3_ fraction (15 and 30 mg/kg, respectively). The values are expressed as means ± SD (*n* = 6). ^##^*p* < 0.01, significant difference for comparison with NC; ^∗^*p* < 0.05, ^∗∗^*p* < 0.01, significant differences for comparison with COPD.

CS is known to induce the accumulation of inflammatory cells such as neutrophils and macrophages in BALF and lung tissue *in vivo* (John et al., 2014). These inflammatory cells stimulate the release of various inflammatory mediators, such as ROS and cytokines (TNF-α and IL-6), resulting in lung inflammation (Shapiro, 1999). Generally, CS and LPS exposure accelerates inflammatory responses in COPD-like *in vivo* models (Moazed et al., 2016) and causes lung injury that is evident in COPD (O’Donnell et al., 2006). For CS- and LPS-exposed (CS/LPS) mice, we investigated whether administration of the CHCl_3_ fraction ameliorates the airway inflammatory responses to CS/LPS exposure. CS/LPS-exposed mice exhibited marked increases in the number of inflammatory cells and in the levels of TNF-α and IL-6 in their BALF (**Figures 2A–C**) when compared with normal controls (NC). By contrast, the CHCl_3_ fraction and roflumilast suppressed these increases in the BALF of CS/LPS-exposed mice. Roflumilast was employed as a positive control because it has been shown to reduce the risk of COPD symptoms in human patients (Rabe et al., 2005). These results were also consistent with the observed histopathology of lung tissues (**Supplementary Figure S1**). CS/LPS-exposed animals showed an accumulation of inflammatory cells in lung tissue, whereas the administration of roflumilast or the CHCl_3_ fraction to CS/LPS-exposed animals suppressed this phenomenon (**Supplementary Figure S1**). Our results support the hypothesis that the CHCl_3_ fraction of Xinyi contains compounds with anti-COPD activity in the murine *in vivo* model as well as *in vitro* in NCI-H292 cells.

### Anti-inflammatory Effects of Seven Lignans Isolated From Xinyi on CS/LPS-Exposed Mice

**FIGURE 3:**
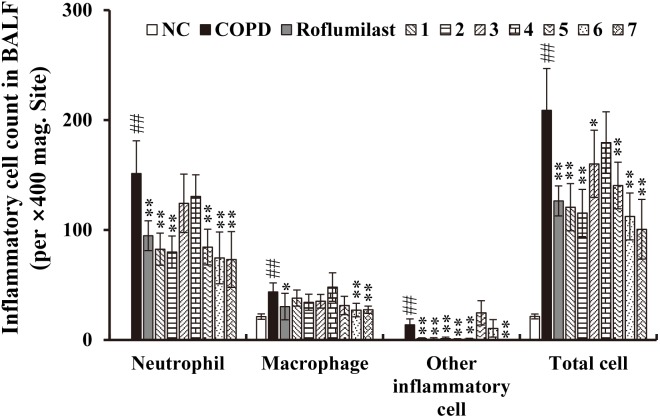
Effects of the seven lignans on the increased number of inflammatory cells in the *in vivo* COPD mouse model. Cells were isolated from BALF and stained with Diff-Quik reagent. Note that the inhibitory effect of all lignans, except lignan 3 and 4, is comparable to that of roflumilast, a known COPD drug. NC, normal control mice; COPD, CS/LPS-exposed mice; Roflumilast, CS/LPS-exposed mice treated with roflumilast (10 mg/kg); 1 to 7, CS/LPS-exposed mice treated with each of the seven lignans (15 mg/kg). The values are expressed as means ± SD (*n* = 6). ^##^*p* < 0.01, significant difference for comparison with NC; ^∗^*p* < 0.05, ^∗∗^*p* < 0.01, significant differences for comparison with COPD.

To identify biologically active compounds from the CHCl_3_ fraction of Xinyi, we performed chromatography and isolated seven lignans as shown in **Supplementary Figure S2**: dimethylpinoresinol (lignan 1), magnolin (lignan 2), dimethyl-liroresinol (lignan 3), epimagnolin (lignan 4), dimethoxyaschantin (lignan 5), aschantin (lignan 6), and fargesin (lignan 7). Next, we tested these lignans for anti-COPD activity, by studying whether their administration suppress airway inflammatory responses in CS/LPS-exposed mice. Except for lignan 4, all isolated lignans, at a dose of 15 mg/kg, significantly inhibited the infiltration of various inflammatory cells, including neutrophils, macrophages, and other leukocytes, into the BALF of CS/LPS-exposed mice (**Figure 3**). Only a small fraction of infiltrating immune cells was marginally affected by lignan 4.

**FIGURE 4:**
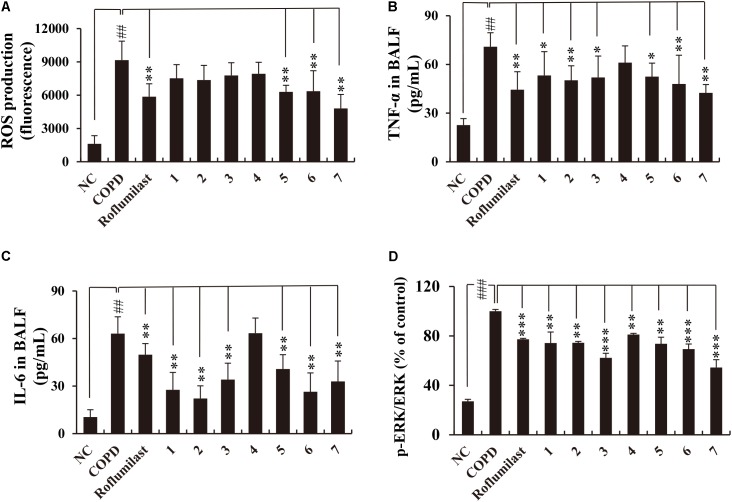
Effects of the seven lignans on the production of ROS, TNF-α, IL-6, and p-ERK in the *in vivo* COPD mouse model. **(A)** ROS production was determined using DCF-DA. **(B–D)** TNF-α, IL-6, and p-ERK levels were determined by ELISA. All lignans except lignan 4 inhibit the production of inflammatory mediators with an efficacy comparable to that of roflumilast. NC, normal control mice; COPD, CS/LPS-exposed mice; Roflumilast, CS/LPS-exposed mice treated with roflumilast (10 mg/kg); 1 to 7, CS/LPS-exposed mice treated with each of the seven lignans (15 mg/kg). Values are expressed as means ± SD (*n* = 6). ^##^*p* < 0.01, significant difference for comparison with NC; ^∗^*p* < 0.05, ^∗∗^*p* < 0.01, and ^∗∗∗^*p* < 0.001, significant differences for comparison with COPD.

Consistent with these data, the accumulation of inflammatory cells in the lung tissue of CS/LPS-exposed animals was also suppressed by the administration of all lignans except lignan 4 (**Supplementary Figure S3**). Moreover, the markedly increased level of ROS production in the BALF obtained from CS/LPS-exposed animals was significantly suppressed by a subset of lignans (5, 6, and 7) (**Figure 4A**). Notably, the highly elevated secretion of TNF-α or IL-6 in the BALF of CS/LPS-exposed animals was effectively reduced by all the isolated lignans, except lignan 4 (**Figures 4B,C**). Interestingly, the efficacy of the lignans was comparable to that of roflumilast (**Figures 3, 4** and **Supplementary Figure S3**), although lignan 4 was an exception.

CS activates ERK signaling, thereby inducing the expression of inflammatory cytokines such as TNF-α or IL-6 (Jeong et al., 2010). In addition, roflumilast exerts its anti-inflammatory effects through inhibiting ERK activation in CS/LPS-exposed animals (Shin et al., 2015; Park et al., 2017). Thus, we checked whether lignans from Xinyi affect p-ERK levels in the lungs of CS/LPS-exposed animals. This test demonstrated that all lignans suppress p-ERK levels in CS/LPS-exposed animals (**Figure 4D**). This suppressive effect of lignans on ERK phosphorylation was comparable to that of roflumilast. Overall, our results indicate that lignans isolated from Xinyi exert anti-inflammatory activity in CS/LPS-exposed mice and that they are biologically active compounds. This makes them promising therapeutic candidates for the treatment of inflammatory airway diseases such as COPD.

### Anti-inflammatory Effects of Lignans on CSC-Stimulated Human Lung Epithelial Cells (NCI-H292)

Since CSC-induced inflammatory response, such as IL-6 expression, has been demonstrated to be associated with the activation of upstream ERK and Akt signaling (Crosbie et al., 2016), we determined whether the isolated lignans (1–7) inhibit CSC-induced IL-6 expression by suppressing the activation of ERK, Akt, or both in NCI-H292 human lung epithelial cells. First, we tested the cytotoxicity of each lignan in NCI-H292 cells at concentrations ranging from 1 to 40 μM. In a culture condition with 0.1% or 10% of FBS, all lignans induced no cytotoxicity below 10 μM (except for only lignan 6 in 10% FBS condition). Therefore, we used this concentration for all subsequent assays (**Supplementary Figures S4, S5**).

**FIGURE 5:**
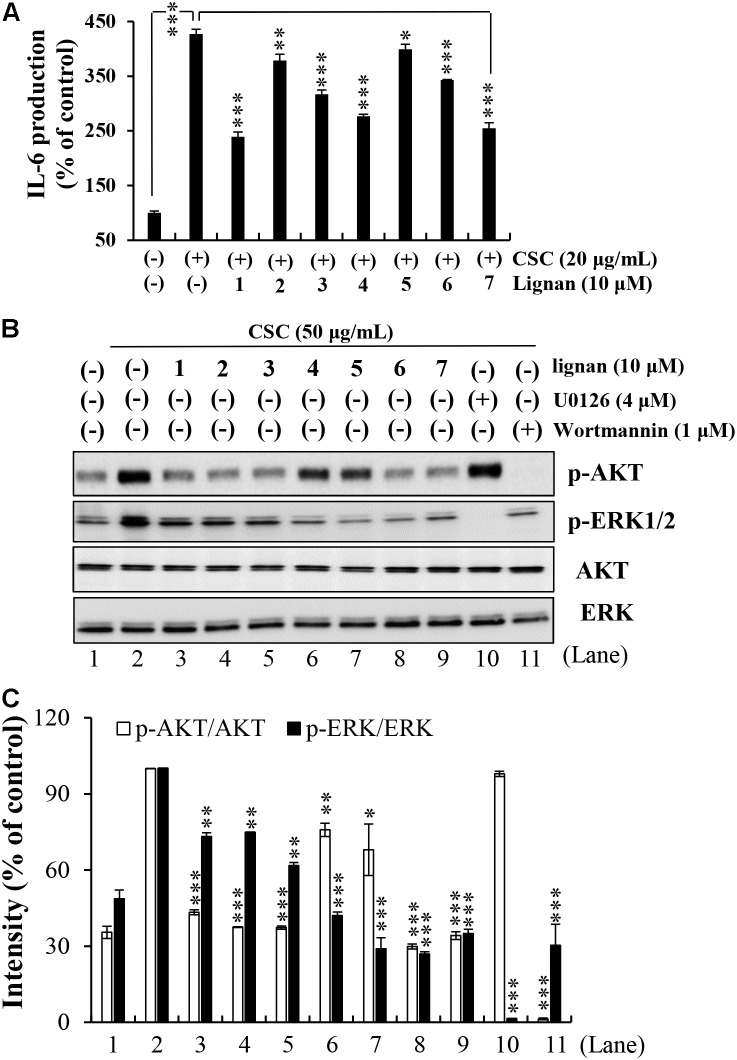
Effects of the seven lignans on CSC-induced Akt and ERK signaling pathways in NCI-H292 cells. **(A)** Effects of the seven lignan compounds on IL-6 levels were assayed by ELISA. NCI-H292 cells were pretreated with individual lignans (10 μM) for 2 h and subsequently treated with CSC (20 μg/mL) for 12 h. Bar graphs represent means ± SD of three independent experiments (^∗^*p* < 0.05, ^∗∗^*p* < 0.01, and ^∗∗∗^*p* < 0.001 for comparison with controls, which were treated with CSC alone). **(B)** NCI-H292 cells were pretreated with individual lignans (10 μM), U0126 (4 μM), or Wortmannin (1 μM) for 2 h and subsequently treated with CSC (50 μg/mL) for 30 min. Total cell lysates were detected by western blotting using anti-p-Akt**, -**Akt, **-**p-ERK1/2, and -ERK1/2 antibodies. The lane numbers are indicated below the western blot image. **(C)** The bar graph represents the percentage intensity of phosphorylated Akt or ERK1/2 normalized to Akt or ERK1/2 levels, respectively. The bar graph presents means ± SD of three independent experiments (^∗^*p* < 0.05, ^∗∗^*p* < 0.01, and ^∗∗∗^*p* < 0.001).

Next, both IL-6 level and phosphorylated ERK and Akt levels following CSC stimulation were investigated in lignan-pretreated NCI-H292 cells. We found that all lignans significantly inhibited the increase in IL-6 protein expression induced by CSC stimulation through the suppression of the ERK and Akt signaling pathways (**Figure 5**). The phosphorylation levels of ERK and Akt were greatly elevated in NCI-H292 cells stimulated by CSC. These increased phosphorylation levels of ERK and Akt were significantly suppressed by each of all seven lignans, simultaneously (**Figures 5B,C**). These results suggested that Xinyi lignans could negatively regulate the upstream signaling elements for ERK or Akt activation.

Interestingly, *in silico* molecular docking studies predicted that two of the lignans (5 and 7) would exhibit high structural and conformational compatibility with the ATP binding site of ERK, as they exhibit the highest energy affinities for the ATP binding site of ERK-2 (bolds in **Supplementary Table S1**). Indeed, a previous study demonstrated that magnolin (lignan 2) targets the active pockets of ERK1 and ERK2 to inhibit cancer cell metastasis (Lee et al., 2015b). Additionally, five of the lignans (1, 3, 5, 6, and 7) also showed high structural affinity for the ATP binding site of Akt-1 (bolds in **Supplementary Table S1**). These *in silico* predictions suggest that the inhibitory action of lignans on IL-6 expression might be also mediated by their direct binding to ERK or Akt.

### Lignan 1 (Dimethylpinoresinol) Inhibits CSC-Induced Inflammatory Response in a Concentration-Dependent Manner in NCI-H292 Cells

Our aforementioned results show that lignan 1 (dimethylpinoresinol) effectively suppresses inflammatory responses in both CSC-stimulated airway epithelial cells and in the CS/LPS-exposed mouse model. This prompted us to take a closer look at the dose-dependent anti-inflammatory effects of lignan 1 on CSC-induced inflammation using human lung epithelial cells.

**FIGURE 6:**
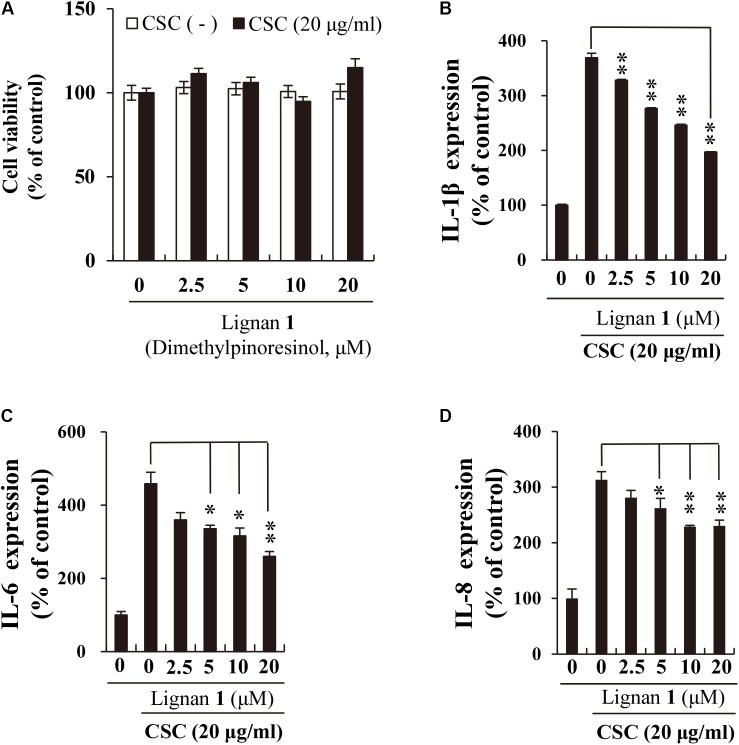
The inhibitory effect of lignan 1 (dimethylpinoresinol) on CSC-induced pro-inflammatory cytokine expression in NCI-H292 cells. **(A)** Lignan 1 had no adverse effects on NCI-H292 cell growth. **(B–D)** The suppressive effect of lignan 1 on the expression of pro-inflammatory cytokines (IL-1β, -6, and -8) was measured by ELISA. NCI-H292 cells were pretreated with various concentrations of lignan 1 for 2 h and subsequently treated with CSC (20 μg/mL) for 12 h. Bar graphs represent means ± SD of three independent experiments (^∗^*p* < 0.05 and ^∗∗^*p* < 0.01, for comparison with controls, which were treated with CSC alone).

Before addressing the suppressive effect of lignan 1 on CSC-induced inflammation in NCI-H292 cells, we assessed the concentration-dependent cytotoxicity of lignan 1 in the absence or presence of CSC for 24 h (**Figure 6A**). Since lignan 1 showed no cytotoxicity at concentrations of up to 20 μM, the subsequent tests were performed at concentrations between 0 and 20 μM.

Next, we investigated the suppressive effects of lignan 1 on CSC-induced expression of cytokines or chemokines, such as IL-1β, IL-6, and IL-8, using ELISA. These inflammatory mediators are well-known protein markers found elevated in COPD patients (Chung, 2001; El-Shimy et al., 2014). Besides reducing IL-6 protein expression, lignan 1 also significantly decreased the expression levels of CSC-induced IL-1β and IL-8 (**Figures 6B–D**). These results suggest that the anti-inflammatory activity of lignan 1 in CSC-stimulated lung epithelial cells may be associated with inhibition of the expression of various cytokine mediators, such as IL-1β, IL-6, and IL-8.

### Lignan 1 Inhibits CSC-Induced Activation of EGFR and Its Downstream Effectors in NCI-H292 Cells

Our results so far indicate that lignan 1 exhibits anti-COPD activity *in vitro* and *in vivo.* However, the pharmacological mechanism underlying the effects of lignan 1 on CSC-induced inflammation remain elusive. We found some clues from our data, which showed that lignan 1 inhibits ERK and Akt phosphorylation/activation (**Figure 5B**), and from previous studies, which reported that CS activates MAPKs (ERK, JNK, and p38) and PI3K/Akt by directly inducing phosphorylation of their upstream regulator, EGFR (Yang et al., 2009; Vallath et al., 2014). Thus, we inferred that lignan 1 might exert its inhibitory effects on ERK or Akt phosphorylation by inhibiting upstream EGFR activation.

To resolve the question of whether lignan 1 has suppressive effects on CSC-induced activation of EGFR and its downstream components, including c-Src, c-Raf, MEK1/2, Akt, and MAPKs, we performed Western blot analysis on total lysates from CSC-stimulated NCI-H292 cells using antibodies specific to the active phosphorylated forms of these proteins.

**FIGURE 7:**
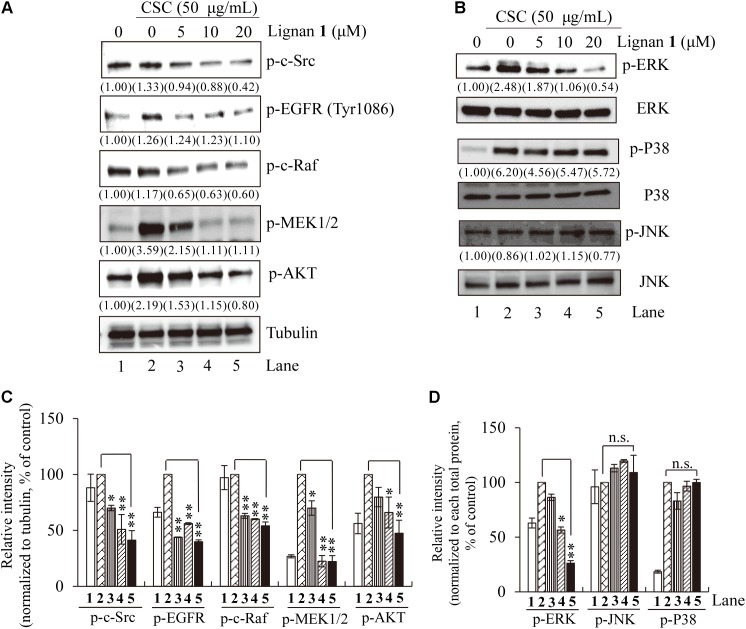
The inhibitory effect of lignan 1 (dimethylpinoresinol) on CSC-activated EGFR and its downstream components. **(A,B)** NCI-H292 cells were pretreated with each concentration of lignan 1 for 2 h and then treated with CSC (50 μg/mL) for 30 min. Total cell lysates were assayed by western blotting using antibodies detecting p-c-Src, p-EGFR, p-c-Raf, p-MEK1/2, p-Akt, p**-**ERK1/2, ERK, p-P38, P38, p-JNK, and JNK. Tubulin was used as a loading control. **(C,D)** Relative band intensities of phosphorylated proteins were normalized to tubulin or total MAPK. The bar graph displays means ± SD of three independent experiments (^∗^*p* < 0.05 and ^∗∗^*p* < 0.01, n.s., not significant).

We found that the phosphorylation level of EGFR is increased by CSC stimulation, and that this CSC-stimulated EGFR activation/phosphorylation is considerably suppressed by lignan 1 pretreatment in a concentration-dependent manner (**Figures 7A,C**). Likewise, except for JNK and p38, CSC-induced phosphorylation of the downstream targets of EGFR – c-Src, c-Raf, MEK1/2, Akt, and ERK – was also significantly reduced by lignan 1 (**Figures 7B,D**), suggesting that lignan 1 may target EGFR itself to suppress the EGFR/ERK/Akt signaling pathway induced by CSC in human lung cells.

**FIGURE 8:**
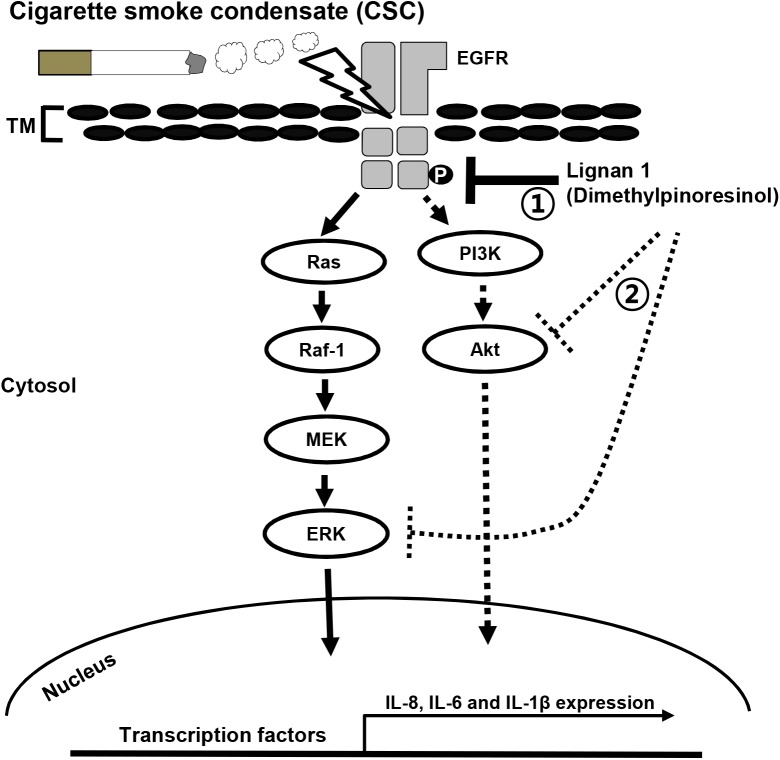
A proposed mechanism for inhibition of the CSC-induced expression of inflammatory cytokines in lung airway epithelial cells by lignan 1. CSC phosphorylates and activates EGFR. This EGFR phosphorylation eventually results in a series of inflammatory reactions in lung epithelial cells. Lignan 1 suppresses the CSC-induced expression of inflammatory cytokines such as IL-8, -6, and -1β, *in vivo* and *in vitro*. Our results suggest that in human airway epithelial cells, the inhibitory action of lignan 1 on cytokine expression may be mediated by reducing phosphorylation/activation on both EGFR and its downstream effectors in the Raf-1/MEK/ERK and PI3K/AKT pathways (number 1). All lignans from Xinyi simultaneously inhibit both ERK and Akt phosphorylations. Based on *in silico* molecular docking study, it could be possible that a direct binding of lignans to ERK or Akt protein might inhibit the enzyme activity of this kinase, even though it is highly speculative hypothesis (number 2). It is uncertain whether lignan 1 directly binds to EGFR itself, its ligands, or its positive regulators to restrain EGFR phosphorylation/activation and subsequent cytokine expression. TM, transmembrane.

Overall, our results indicate that the anti-inflammatory activity of lignan 1 and other lignans isolated from Xinyi in NCI-H292 cells may be mediated through the suppression of CSC-stimulated EGFR signaling, including inhibition of its downstream effectors, ERK and Akt (**Figure 8**).

## Discussion

In the treatment of COPD, ICSs are used as an effective medication. However, ICSs have some limitations, including side effects such as pneumonia and osteoporosis. Thus, there is growing awareness of the importance of medicinal plants in treating COPD symptoms in safe and more efficacious ways (Ekor, 2014). Currently, various herbal remedies have been considered as good alternatives or as complementary to standard therapies for lung inflammatory diseases such as lung cancer (Gao et al., 2016), asthma (Park et al., 2012), and COPD (Luo et al., 2015). Indeed, the addition of purified Xinyi extracts to ICSs is used to treat inflammatory diseases such as asthma and COPD, and this approach has had beneficial effects, such as good tolerability and fewer side effects, in asthma patients (Park et al., 2012). However, the exact components in Xinyi extracts that exert therapeutic effects in COPD patients and their functions had not yet been well established.

In this study, for the first time, we isolated seven lignan compounds from the active CHCl_3_ fraction of Xinyi and showed that these components possess effective anti-inflammatory activity in both CSC-stimulated airway epithelial cells and in an *in vivo* COPD-like animal model established by the exposure of mice to CS and LPS.

Lung inflammation in COPD patients is worsened by exposure to CS (Lugade et al., 2014). Indeed, in the BALF from cigarette smokers, both inflammatory mediators (ROS, TNF-α, and IL-6) and the numbers of inflammatory cells (neutrophils, eosinophils, and macrophages) are increased compared to their values in the BALF of non-smokers (O’Donnell et al., 2006). Thus, it is important to understand the mechanisms underlying the inflammatory responses induced by CS exposure for improved treatment of airway inflammatory diseases such as COPD (Lee et al., 2012). In the lung of COPD patients, the cytokine IL-6 is suggested as one of the main inducers causing lung fibrosis and lung tissue remodeling. Interestingly, the previous studies using the mice models of pulmonary fibrosis showed that inhibition of IL-6 signaling attenuates lung fibrosis (Salazar and Herrera, 2011; Le et al., 2014; Kobayashi et al., 2015; Lin and Jiang, 2015). Thus, blockage of IL-6 signaling could be a possible therapeutic way for attenuating or relieving pathological symptoms of COPD patients. Our results reveal that CSC-induced IL-6 expression in human airway epithelial cells was significantly inhibited by the CHCl_3_ fraction extracted from Xinyi. Moreover, this active fraction also suppressed the infiltration of inflammatory cells (neutrophils, macrophages, and lymphocytes) and the production of inflammatory mediators such as ROS, TNF-α, and IL-6 in a COPD-like mouse model. These results suggest that the CHCl_3_ fraction extracted from Xinyi contains compounds that counter lung inflammation in diseases such as COPD.

For further investigation, we isolated seven lignans from the active fraction of Xinyi and investigated their anti-inflammatory activities in a COPD-like mouse model. Lignans are an important class of active, naturally occurring plant compounds, exhibiting anti-inflammatory (Bastos et al., 2001), anti-tumorigenic, anti-mitotic, anti-viral, and anti-atherosclerotic activities (MacRae and Towers, 1984). We studied whether these seven lignans (1–7) could be used as therapeutic agents to manage airway inflammatory diseases such as COPD. Our results show that a subset of these lignans effectively decreased the infiltration of neutrophils and macrophage cells, as well as the production of inflammatory mediators such as ROS and cytokines (TNF-α and IL-6). Interestingly, the efficacies of all isolated lignans, except lignan 4, are comparable to that of roflumilast, a commercialized COPD drug. These data strongly support the hypothesis that the active fraction from Xinyi and the lignans isolated from it could potentially be used to manage lung inflammatory diseases, including COPD.

It is well-known that CS induces ligand-independent phosphorylation of EGFR (Mueller et al., 2008), leading to subsequent activation of the MEK/ERK (Mercer and D’Armiento, 2006), and PI3K/Akt pathways (Yang et al., 2009). These signaling pathways have been shown to be involved in lung inflammatory diseases such as COPD (Geraghty et al., 2014). Indeed, PD98059 (an inhibitor of MEK/ERK) and LY294002 (a non-selective inhibitor of PI3K) have been reported to suppress CS-induced cytokine expression in airway epithelial cells (Hellermann et al., 2002). In particular, theophylline (a selective inhibitor of PI3K-δ used as a pharmacological treatment option for COPD) inhibits CS-induced oxidative stress to reverse corticosteroid insensitivity in COPD patients (To et al., 2010).

Our results show that all seven lignan compounds significantly reduced IL-6 protein levels that were elevated in response to CSC in NCI-H292 cells. In case of all seven lignan compounds, this was achieved through the suppression of phosphorylated ERK and AKT levels. In previous studies, it was reported that magnolin (lignan 2) inhibits cell migration and invasion by targeting the ERKs/RSK2 signaling pathway (Lee et al., 2015b) and that aschantin (lignan 6) inhibits EGF-induced activation of Akt and subsequent Akt-mediated GSK3β phosphorylation at Ser9 (Lee et al., 2015a). Our results seem to be in the same line with those of these previous reports.

Additionally, our *in silico* molecular docking study show an interesting possibility: Xinyi lignans could directly bind to the ATP binding site of ERK or Akt to inhibit its enzymatic activity. Although it is highly speculative and needed to be tested by *in vitro* kinase assay, this prediction suggests that there might be another mechanism of action for lignans to inhibit airway inflammation.

In COPD and lung cancer, CS is known to be a common causative agent. As a proposed mechanistic model in mice, it is suggested that CS may activate the EGFR signaling cascade, which includes ERK and Akt, in a ligand-independent manner. In this model, first, CS induces c-Src phosphorylation and ligand-independent EGFR phosphorylation. These activations result in the phosphorylation of subsequent downstream effectors in Raf/MEK/ERK or PI3K/Akt signaling pathways, eventually leading to lung tissue destruction by airway inflammation (Geraghty et al., 2014). This CS-induced airway inflammation and the destructive changes in the lungs of smoke-exposed mice can be ameliorated by AZD0530, a c-Src inhibitor (Khan et al., 2008; Geraghty et al., 2014). Consistent with this previous report, we demonstrated that one of our lignan compounds, lignan 1 (dimethylpinoresinol), suppresses CS-induced inflammatory responses by suppressing the phosphorylation of c-Src, EGFR, c-Raf, MEK, ERK, and Akt. These data strongly suggest that the lignans in Xinyi may inhibit inflammatory response through inactivation of EGFR signaling, leading to ERK or PI3K/Akt activation (**Figure 8**).

To the best of our knowledge, we have, for the first time, established that seven lignan compounds isolated from the active fraction of Xinyi exert anti-COPD effects through the effective suppression of CS/LPS-induced inflammatory responses *in vitro* and *in vivo*. Therefore, we suggest that the combined use of active lignans isolated from Xinyi is a promising therapeutic modality for treating lung inflammatory diseases, including COPD and lung cancer.

## Author Contributions

S-UL and HR conceived and designed the experiments. SL, I-SS, J-HC, J-WL, and JL performed the experiments. MOK, H-JL, and K-SA analyzed the data. S-TH and S-RO wrote the paper. All authors were involved in writing and critical review of the manuscript and approved the final version.

## Conflict of Interest Statement

The authors declare that the research was conducted in the absence of any commercial or financial relationships that could be construed as a potential conflict of interest.

## Footnotes

1 http://www.jhanbang.co.kr/

## Abbreviations

BALF: bronchoalveolar lavage fluid
COPD: chronic obstructive pulmonary disease
CS: cigarette smoke
CSC: cigarette smoke condensate
DCF-DA: 2′,7′-dichlorofluorescin diacetate
GM: growth medium
ICS: inhaled corticosteroid
LPS: lipopolysaccharides
MEK: MAPK kinase
RSK2: ribosomal S6 kinase 2
