# Supplementary material for: Lignans Isolated From Flower Buds of Magnolia fargesii Attenuate Airway Inflammation Induced by Cigarette Smoke in vitro and in vivo
Source: Front Pharmacol. 2018 Sep 7;9:970. doi: 10.3389/fphar.2018.00970 (PMC6143820; doi:10.3389/fphar.2018.00970)
Supplement: Supplementary file 1 [file Data_Sheet_1.PDF]

Supplementary Information

Lignans isolated from flower buds of *Magnolia fargesii*

attenuate airway inflammation induced by cigarette smoke *in vitro* and *in vivo*

● Supplementary Methods

● Supplementary Figures S1-5

Figure S1. Airway inflammation in lung tissue from mice exposed to both CS and LPS was ameliorated by the $CHCl_3$ fraction.

Figure S2. Schematic diagram illustrating the isolation of seven lignans (**1**-**7**) present in *Magnolia fargesii* extracts by chromatography.

Figure S3. Airway inflammation in lung tissue of mice exposed to CS and LPS was ameliorated by the seven lignans (**1**-**7**).

Figure S4. The seven lignans isolated from Xinyi have no cytotoxicity at concentrations below 10 μM.

Figure S5. The seven lignans isolated from Xinyi at concentrations below 10 μM have no cytotoxicity in a condition of high cell proliferation except for lignan 6.

Table S1. Molecular docking study for MEK1/2, ERK1/2, or AKT1 with seven lignans (**1**-**7**).

● Supplementary Table

- **Table S1.** Molecular docking study of MEK1/2, ERK1/2 or AKT1 with seven lignans (**1**-**7**).

Method for Fig. S1

Histological examination

After the BALF samples were obtained, lung tissue was fixed in 10% (v/v) neutral buffered formalin. The tissues were embedded in paraffin, sectioned to 4-μm thickness, and stained with hematoxylin and eosin solution to allow estimation of the inflammatory response. Photomicrographs were obtained using a Photometric Quantix digital camera, and montages were assembled in Adobe Photoshop7.0. The images were adjusted for brightness and contrast, but were not otherwise manipulated.

Fig. S1

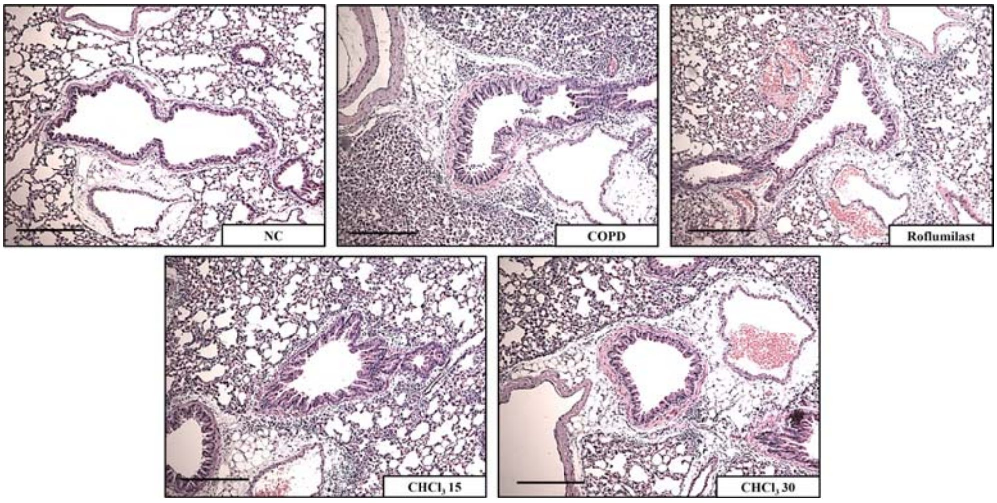

Figure S1. Airway inflammation in lung tissue from mice exposed to both CS and LPS was ameliorated by the $CHCl_3$ fraction. Representative figures of H&E staining of lung tissue. In COPD mice, inflammatory cells were accumulated in lung tissue after CS/LPS exposure. This accumulation of immune cells was reduced by pretreatment of mice with roflumilast or the CHCl3 fraction from Xinyi. NC: normal control mice; COPD: CS/LPS-exposed mice; Roflumilast: CS/LPS-exposed mice treated with roflumilast (10 mg/kg); $CHCl_3$ (15 and 30 mg/kg, respectively); CS/LPS exposed mice treated with the $CHCl_3$ fraction (15 and 30 mg/kg, respectively). Scale bars, 200 μm.

Method for Fig. S2 and S3

The air-dried flower buds (8 kg) of *Magnolia fargesii* were extracted with MeOH (5 L × 3) at room temperature. The combined extracts were concentrated *in vacuo* to yield a brown resin (1.2 kg), which was partitioned with *n*-hexane, $CHCl_3$, *n*-BuOH, and H_2O. The chloroform layer was washed with brine, dried over anhydrous Na_2SO_4, and then concentrated to give a viscous residue (140 g). The $CHCl_3$ fraction was subjected to chromatography on a silica gel column and eluted using a gradient of 100 % hexane to 100 % ethyl acetate. Thirteen pooled fractions (Fr.1-Fr.13) were obtained after combining fractions with similar TLC profiles from this initial column chromatography. The fractions Fr.10-12 (16.9 g) were resolved by chromatography over a silica gel as the stationary phase using an *n*-hexane-EtOAc gradient (from 10:1 to 1:1 v/v) as the mobile phase to afford 5 fractions (A01-A5). Of these, fraction A03 (14.6 g) was separated by chromatography on a normal-phase (NP) silica gel column (5 × 60 cm, YMC-DispoPack SIL, 500 g) using MPLC (KeyChem Flash, YMC Korea co., Ltd) with a stepwise gradient of $CHCl_3$/ethyl acetate (0-20 min, 0% EtOAc; 20-40 min, 5% EtOAc; 40-120 min, 10% EtOAc) and a flow rate of 20 mL/min to give dimethoxyaschantin **5** (696.0 mg), aschantin **6** (339.0 mg), and fargesin **7** (386.0 mg). The fraction Fr.13 (28.5 g) was resolved by chromatography over a silica gel as stationary phase using an *n*-hexane-EtOAc gradient (from 8:1 to 1:1 v/v) as the mobile phase to yield 5 fractions (B01-B05). Sub-fraction B04 (1.71 g), enriched with compound **3** was purified on an NP silica gel column (5 × 60 cm, YMC-DispoPack SIL, 500 g) and eluted with $CHCl_3$/acetone/MeOH to afford dimethylliroresinol **3** (171.0 mg). Sub-fraction B01 (15.0 g), enriched with **1**, **2**, and **4**, was purified on an NP silica gel column (YMC GEL SIL-HG 20 mm, 220 g) and eluted with *n*-hexane/$CHCl_3$/EtOAc = 5:2:1 to afford magonolin **2** (628.0 mg) and a mixture of **1** and **4** (Sub-fraction B11). Of these, fractions B11 (4.2 g) was subjected to chromatography by prep-HPLC using a reversed-phase column (YMC–Pack ODS-AQ-HG, 10 mm) and eluted with MeOH−H_2O isocratic (55% methanol, 40 min) by repeated injection of 400 μL (100 mg/mL MeOH dilutions) to give dimethylpinoresinol **1** (214.0 mg) and epimagnolin **4** (146.0 mg).

Fig. S2

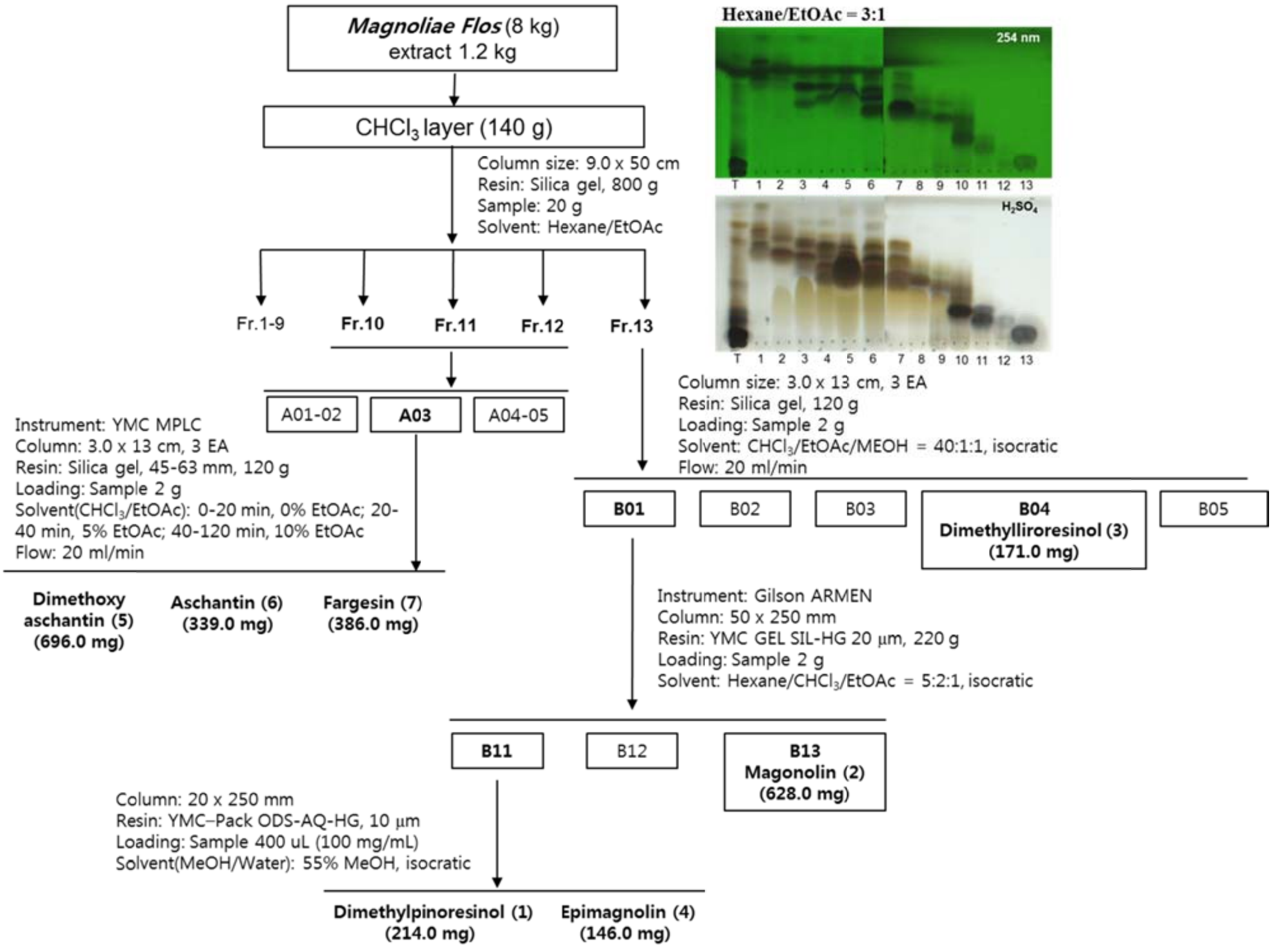

Figure S2A. Schematic diagram illustrating the isolation of seven lignans (**1**-**7**) present in *Magnolia fargesii* extracts by chromatography.

<table>
<tr><td colspan="2" rowspan="6">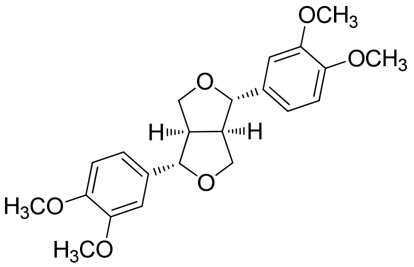</td><td>Sample Name</td><td>Dimethylpinoresinol (**1**)</td></tr>
<tr><td>Physical data</td><td>Amorphous solid, $[\alpha]_D$ +55.0 ($CHCl_3$, *c* 2.4)</td></tr>
<tr><td>MW</td><td>386</td></tr>
<tr><td>mp</td><td></td></tr>
<tr><td>UV</td><td>238, 278 nm</td></tr>
<tr><td>HRESIMS $[M+H]^+$</td><td>387.1809 (calcd for C22H27O6 387.1808)</td></tr>
<tr><td>^{1}H NMR ($CDCl_3$, 500 MHz)</td><td colspan="3">3.12 (2H, m, H-1/H-5), 3.87 (6H, s, 3', 3"-OMe), 3.88 (2H, m, H-4a/H-8a), 3.90 (6H, s, 4', 4"-OMe), 4.26 (2H, m, H-4b/H-8b), 4.76 (2H, d, *J* = 4.3 Hz, H-2/H-6), 6.85 (2H, d, *J* = 8.0 Hz, H-5'/H-5"), 6.89 (2H, dd, *J* = 2.0, 8.5 Hz, H-6'/H-6"), 6.91 (2H, d, *J* = 2.0 Hz, H-2'/H-2")</td></tr>
<tr><td>^{13}C NMR ($CDCl_3$, 120 MHz)</td><td colspan="3">54.2 (C-1/C-5), 55.9 (OMe, C-3'/C-4'/C-3"/C-4"), 71.7 (C-4/C-8), 85.8 (C-2/C-6), 109.3 (C-2'/C-2"), 111.1 (C-5'/C-5"), 118.3 (C-6'/C-6"), 133.6 (C-1'/C-1"), 148.6 (C-4'/C-4"), 149.2 (C-3'/C-3")</td></tr>
</table>

<table>
<tr><td colspan="2" rowspan="6">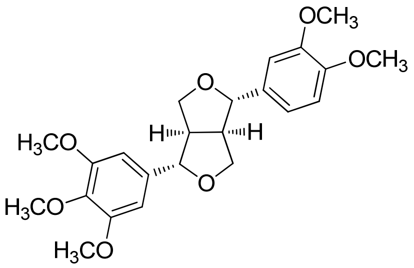</td><td>Sample Name</td><td>Magnolin (**2**)</td></tr>
<tr><td>Physical data</td><td>viscous oil, $[\alpha]_D$ +44.0 ($CHCl_3$, *c* 2.0)</td></tr>
<tr><td>MW</td><td>416</td></tr>
<tr><td>mp</td><td></td></tr>
<tr><td>UV</td><td>234, 277 nm</td></tr>
<tr><td>HRESIMS $[M+H]^+$</td><td>417.1884 (calcd for C23H28O7 417.1913)</td></tr>
<tr><td>^{1}H NMR ($CDCl_3$, 500 MHz)</td><td colspan="3">3.11 (2H, m, H-1/H-5), 3.83 (6H, d, *J* = 1.5 Hz, OMe), 3.87 (6H, d, *J* = 1.0 Hz, OMe), 3.90 (3H, d, *J* = 1.5 Hz, OMe), 3.92 (2H, dd, *J* = 3.5, 9.0 Hz, H-4a/H-8a), 4.28 (2H, m, H-4b/H-8b), 4.76 (2H, dd, *J* = 4.0, 10.5 Hz, H-2/H-6), 6.57 (2H, s, H-2'/H-6'), 6.85-6.91 (3H, m, Aromatic H, H-5"/H-6"/H-2")</td></tr>
<tr><td>^{13}C NMR ($CDCl_3$, 120 MHz)</td><td colspan="3">54.1 (C-5), 54.4 (C-1), 55.9 (OMe, C-3"), 56.0(OMe, C-4"), 56.2 (OMe, C-3'/C-5'), 60.9 (OMe, C-4'), 71.8 (C-8), 72.0 (C-4), 85.7 (C-6), 86.0 (C-2), 102.8 (C-2'/C-6'), 109.2 (C-2"), 111.1 (C-5"), 118.2 (C-6"), 133.5 (C-1"), 136.8 (C-1'), 137.5 (C-4'), 148.7 (C-4"), 149.2 (C-3"), 153.4 (C-3'/C-5')</td></tr>
</table>

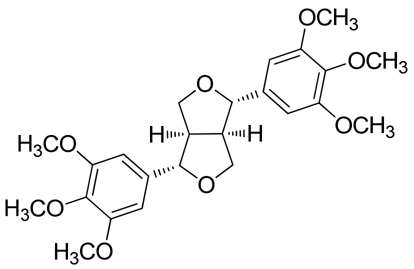

Sample Name	dimethyl liroresinol (**3**)
Physical data	amorphous solid; $[\alpha]_D$ +42.9 ($CHCl_3$, *c* 1.0)
MW	446
mp	
UV	238, 270 nm
HRESIMS $[M+H]^+$	447.2017 (calcd for C24H31O8 447.2017)

^{1}H NMR ($CDCl_3$, 500 MHz)	3.10 (2H, m, H-1/H-5), 3.83 (6H, s, 4', 4''-OMe), 3.87 (12H, s, 3', 3'', 5', 5''-OMe), 3.94 (2H, dd, *J* = 3.6, 9.2 Hz, H-4a/H-8a), 4.32 (2H, m, H-4b/H-8b), 4.75 (2H, d, *J* = 4.3 Hz, H-2/H-6), 6.57 (4H, s, ArH)
^{13}C NMR ($CDCl_3$, 120 MHz)	54.4 (C-1/C-5), 56.3 (3', 3'', 5', 5''-OMe), 60.9 (4', 4''-OMe), 72.0 (C-4/C-8), 86.0 (C-2/C-6), 103.0 (C-2'/C-2''/C-6'/C-6''), 136.8 (C-1'/C-1''), 137.7 (C-4'/C-4''), 153.5 (C-3'/C-3''/ C-5'/C-5'')

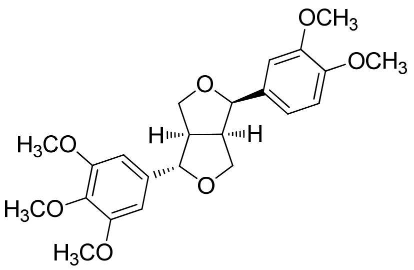

Sample Name	Epimagnolin (**4**)
Physical data	colorless oil, $[\alpha]_D$ +88.4 (MeOH, *c* 0.25)
MW	416
mp	
UV	234, 277 nm
HRESIMS $[M+H]^+$	417.1914 (calcd for C23H28O7 417.1913)

^{1}H NMR ($CDCl_3$, 400 MHz)	2.93 (1H, m, H-5), 3.34 (2H, m, H-1/H-8a), 3.88 (2H, m, H-4a/H-8b), 3.85, 3.89, 3.90, 3.90, 3.93 (OMe), 4.16 (1H, d, J = 9.5 Hz, H-4b), 4.45 (1H, d, J = 7.2 Hz, H-6), 4.90 (1H, d, J = 5.5 Hz, H-2), 6.61 (2H, s, H-2''/H-6''), 6.88 (2H, s, H-5'/H-6'), 6.95 (1H, s, H-2')
^{13}C NMR ($CDCl_3$, 100 MHz)	50.5 (C-1), 55.0 (C-5), 56.3, 56.3, 56.6, 61.3 (3', 4', 5', 3'', 4''-OMe), 70.2 (C-8), 71.4 (C-4), 82.4 (C-2), 88.2 (C-6), 103.3 (C-2''/C-6''), 109.3 (C-2'), 111.4 (C-5'), 118.1 (C-6'), 131.3 (C-1'), 137.3 (C-1''), 137.9 (C-4''), 148.4 (C-4'), 149.2 (C-3'), 153.8 (C-3''/C-5'')

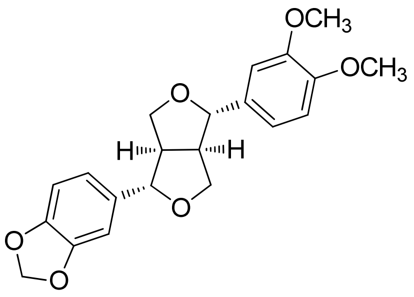	Sample Name	Dimethoxyaschantin (**5**)
	Physical data	colorless oil, $[\alpha]_D$ +59.5 ($CHCl_3$, *c* 2.13)
	MW	370
	mp	
	UV	234, 283 nm
	HRESIMS $[M+H]^+$	371.1491 (calcd for C21H23O6 371.1495)
^1H NMR ($CDCl_3$, 500 MHz)	3.08 (2H, m, H-1/H-5), 3.88 (3H, s, 4'-OMe), 3.86-3.89 (2H, m, overlapped with OMe, H-4a/H-8a), 3.90 (3H, s, 3'-OMe), 4.25 (2H, m, H-4b/H-8b), 4.74 (2H, t, *J* = 5.5 Hz, H-2/H-6), 5.95 (2H, s, -OCH_2O-), 6.77-6.91 (6H, m, ArH, H-2'/H-2"/H-5'/H-5"/H-6'/H-6")	
^{13}C NMR ($CDCl_3$, 120 MHz)	54.2 (C-1), 54.3 (C-5), 55.9(4'-OMe), 56.0 (3'-OMe), 71.7 (C-8), 71.8 (C-4), 85.76, 85.82 (C-2/C-6) 101.1 (-OCH2O-), 106.5(C-2"), 108.2 (C-5"), 109.2 (C-2'), 111.1 (C-5'), 118.3 (C-6'), 119.4 (C-6"), 133.5 (C-1'), 135.1 (C-1"), 147.1 (C-4"), 148.0 (C-3"), 148.6 (C-4'), 149.2 (C-3')	

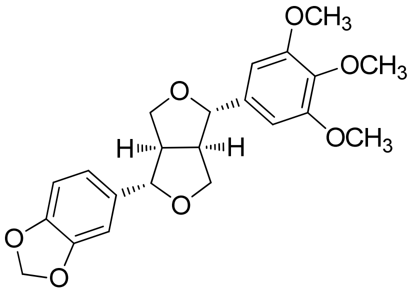	Sample Name	Aschantin (**6**)
	Physical data	viscous oil, $[\alpha]_D$ +49.0 ($CHCl_3$, *c* 1.33)
	MW	400
	mp	
	UV	234, 283 nm
	HRESIMS $[M+H]^+$	401.1578 (calcd for C22H25O7 401.1600)
^1H NMR ($CDCl_3$, 500 MHz)	3.08 (2H, m, H-1/H-5), 3.84 (3H, s, 4'-OMe), 3.88 (6H, s, 3', 5'-OMe), 3.89 (1H, dd, J = 4.0, 9.0 Hz, H-4a), 3.91 (1H, dd, J = 4.0, 9.0 Hz, H-8a), 4.26 (1H, dd, J = 6.5, 9.0 Hz, H-4b), 4.29 (1H, dd, J = 6.5, 9.0 Hz, H-8b), 4.73 (2H, t, J = 4.4 Hz, H-2/H-6), 5.95 (2H, s, -OCH2O-), 6.57 (2H, s, H-2'/H-6'), 6.79 (1H, d, J = 8.0 Hz, H-5"), 6.80 (1H, dd, J = 1.5, 8.0 Hz, H-6"), 6.85 (1H, d, J = 1.5 Hz, H-2")	
^{13}C NMR ($CDCl_3$, 120 MHz)	54.3 (C-5), 54.4 (C-1), 56.2 (3', 5'-OMe), 60.9 (4'-OMe), 71.7 (C-4), 72.0 (C-8), 85.8 (C-6), 86.0 (C-2), 101.1 (-OCH_2O-), 102.8 (C-2'/C-6'), 106.5 (C-2"), 108.2 (C-5"), 119.4 (C-6"), 135.0 (C-1"), 136.8 (C-1'), 137.5 (C-4'), 147.1 (C-4"), 148.0 (C-3"), 153.4 (C-3'/C-5')	

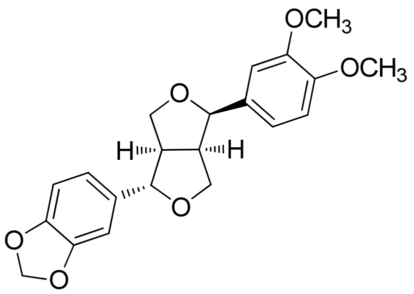

	Sample Name	Fargesin (**7**)
	Physical data	white powder, $[\alpha]_D$ +93.5 ($CHCl_3$, *c* 0.6)
	MW	370
	mp	136-139 °C
	UV	234, 282 nm
	HRESIMS $[M+H]^+$	371.1467 (calcd for C21H23O6 371.1495)
^{1}H NMR ($CDCl_3$, 500 MHz)	2.88 (1H, m, H-5), 3.28-3.35 (2H, m, H-8a/H-1), 3.82-3.86 (2H, m, H-8b/H-4a), 3,88 (3H, s, OMe), 3.90 (3H, s, OMe), 4.13 (1H, dd, *J* = 0.7, 9.4 Hz, H-4b), 4.43 (1H, d, *J* = 7.0 Hz, H-6), 4.87 (1H, d, *J* = 5.0 Hz, H-2), 5.95 (2H, dd, *J* = 1.5, 2.3 Hz, $-OCH_2O-$, H-3"/H-4"), 6.76-6.87 (5H, m ArH), 6.93 (1H, s, ArH)	
^{13}C NMR ($CDCl_3$, 120 MHz)	50.2 (C-1), 54.6 (C-5), 55.91 (OMe, C-3'), 55.94 (OMe, C-4'), 69.8 (C-8), 71.0 (C-4), 82.0 (C-2), 87.7 (C-6), 101.0 (-OCH2O-, C-3"/C-4"), 106.5 (C-2"), 108.2 (C-2'), 109.0 (C-5'), 111.1 (C-5"), 117.7 (C-6"), 119.6 (C-6'), 130.9 (C-1"), 135.2 (C-1'), 147.2 (C-3"), 148.0 (C-4"), 148.0 (C-3'), 148.9 (C-4')	

UPLC-QTof-MS analysis

Magnolia fargesii fraction profiling was performed using an ACQUITY UPLCTM system (Waters Corporation, Milford, MA, USA) equipped with a binary solvent delivery manager and a sample manager coupled to a Micromass Q-TOF Premier^{TM} mass spectrometer (Waters Corporation) with an electrospray ionisation (ESI) interface with MassLynex V4.1 software. Chromatographic separation was performed using an ACQUITY HSS T3 chromatography column (2.1 × 100 mm, 1.8 μm). The column temperature was maintained at 35°C, and the mobile phases A and B were water with 0.1% formic acid and acetonitrile with 0.1% formic acid, respectively. The gradient elution program was as follows: 0.0-1.0 min, 10% B; 1.0-12.0 min, 10-98% B; wash for 1.4 min with 100% B; and a 1.6 min recycle time. The injection volume was 1.0 μL, and the flow rate was 0.4 mL/min. The mass spectrometer was operated in positive ion mode. N_2 was used as the desolvation gas. The desolvation temperature was 350°C, the flow rate was 500 L/h, and the source temperature was 100°C. The capillary and cone voltages were 2700 V and 27 V, respectively. The data were collected for each test sample from 200 to 1500 Da with 0.25-s scan time and 0.01-s interscan delay over the 25 min analysis time. Leucine-enkephalin was used as the reference compound (m/z 556.2771 in the positive mode).

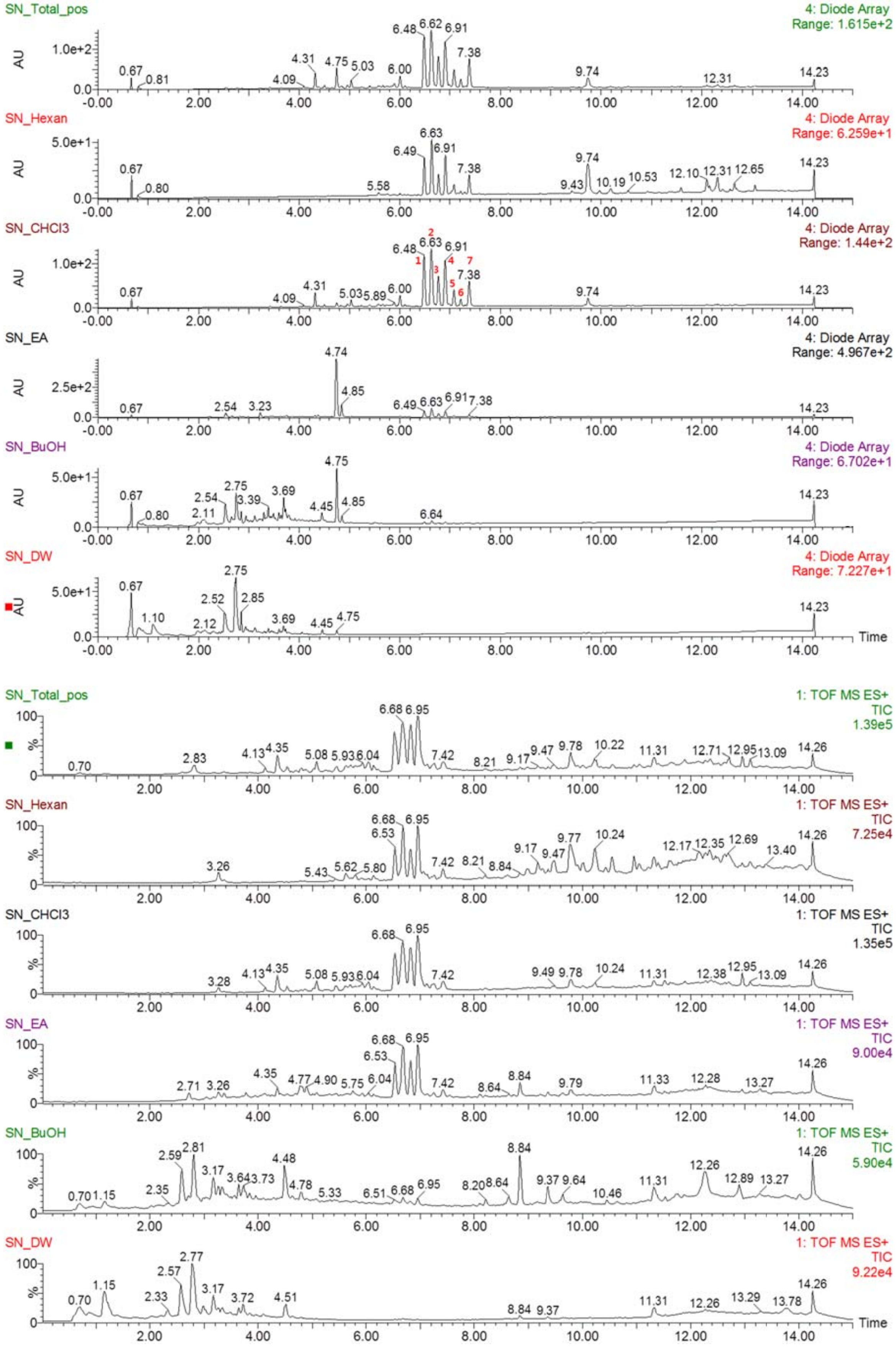

Figure S2B. UPLC-QTof-MS chromatogram of methanolic extract and solvent fraction.

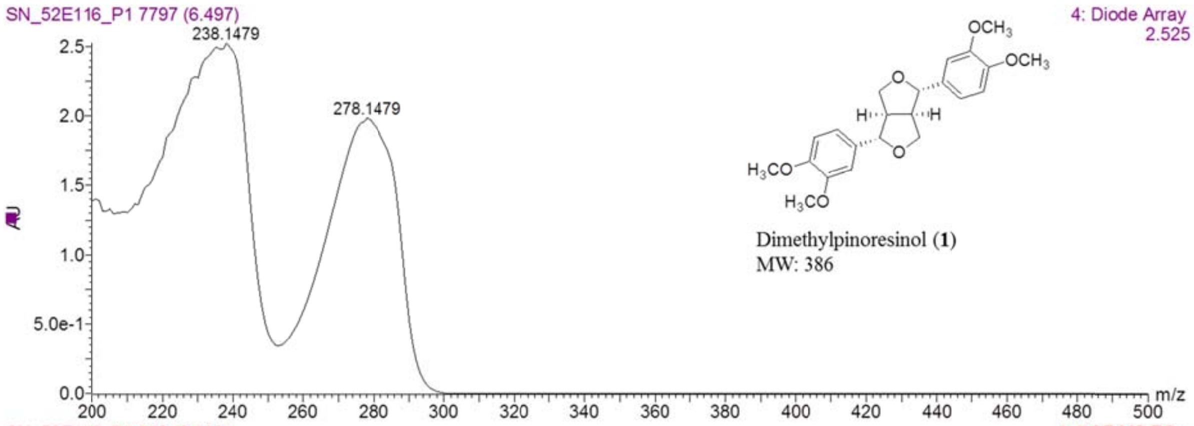

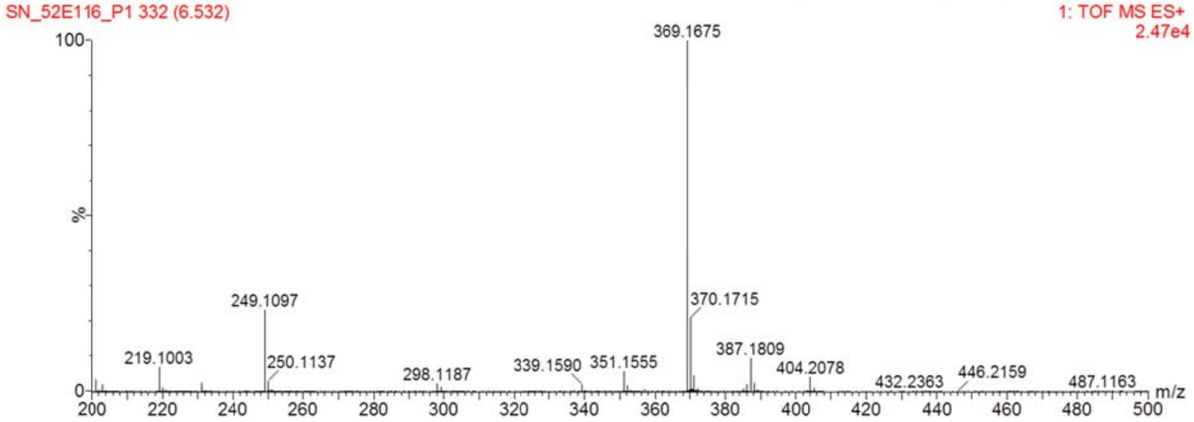

Single Mass Analysis
Tolerance = 10.0 PPM / DBE: min = -1.5, max = 50.0
Element prediction: Off
Number of isotope peaks used for i-FIT = 3
Monoisotopic Mass, Even Electron Ions
86 formula(e) evaluated with 1 results within limits (up to 50 best isotopic matches for each mass)
Elements Used:

Mass	Calc. Mass	mDa	PPM	DBE	Formula	i-FIT	i-FIT Norm	Fit Conf %	C	H	O
387.1809	387.1808	0.1	0.3	9.5	C22 H27 O6	100.0	n/a	n/a	22	27	6

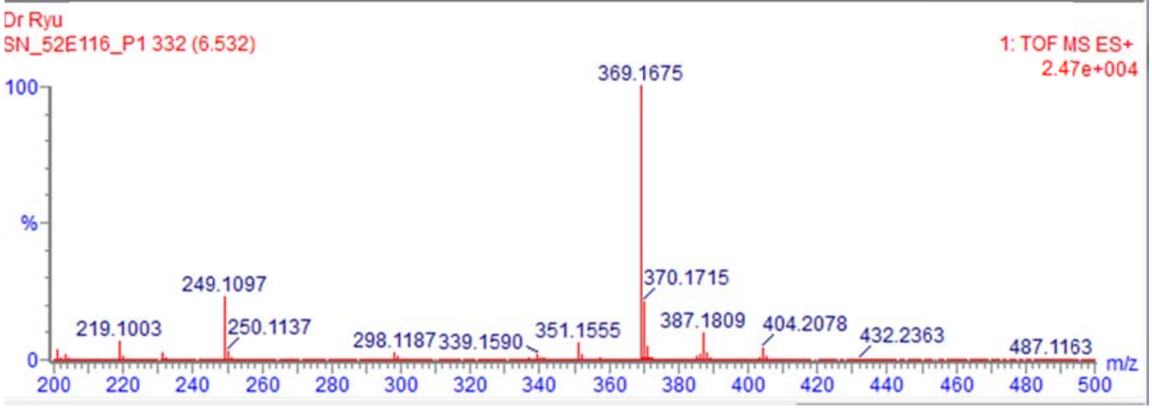

Figure S2C. UV, MS/MS, MS and HREIMS data of lignan **1**.

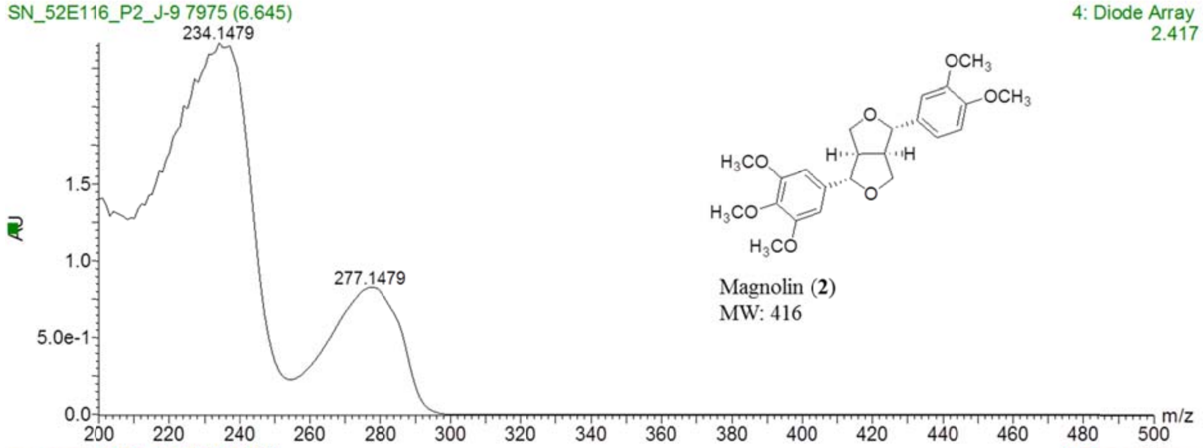

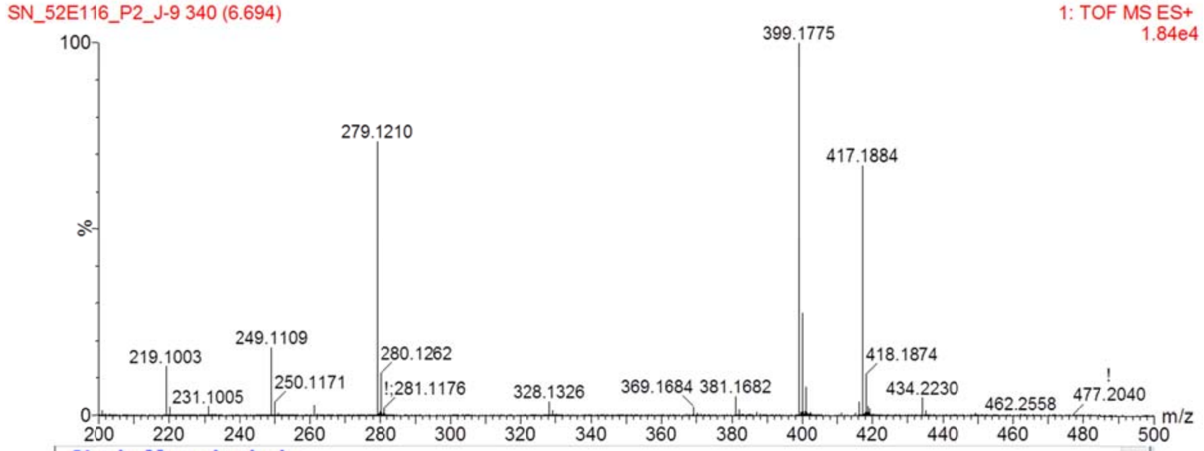

Single Mass Analysis

Tolerance = 10.0 PPM / DBE: min = -1.5, max = 50.0

Element prediction: Off

Number of isotope peaks used for i-FIT = 3

Monoisotopic Mass, Even Electron Ions

93 formula(e) evaluated with 2 results within limits (up to 50 best isotopic matches for each mass)

Elements Used:

Mass	Calc. Mass	mDa	PPM	DBE	Formula	i-FIT	i-FIT Norm	Fit Conf %	C	H	O
417.1884	417.1913	-2.9	-7.0	9.5	C23 H29 O7	147.2	0.162	85.04	23	29	7
	417.1855	2.9	7.0	18.5	C30 H25 O2	148.9	1.900	14.96	30	25	2

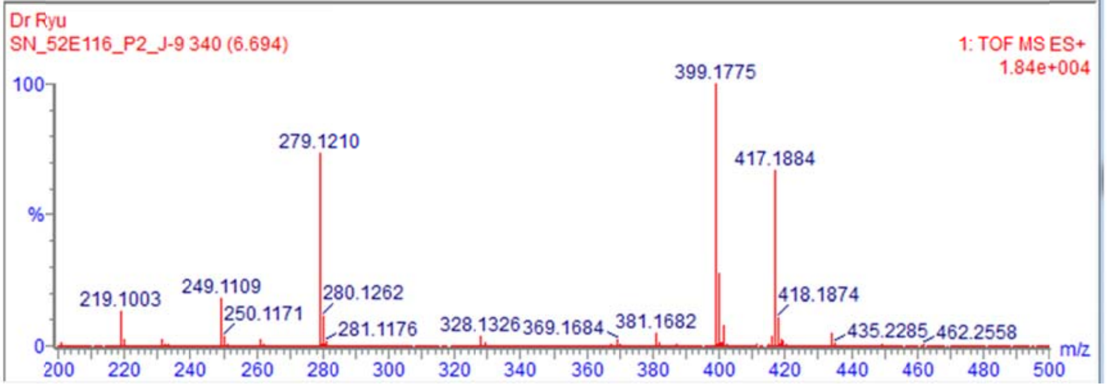

Figure S2D. UV, MS/MS, MS and HREIMS data of lignan **2**.

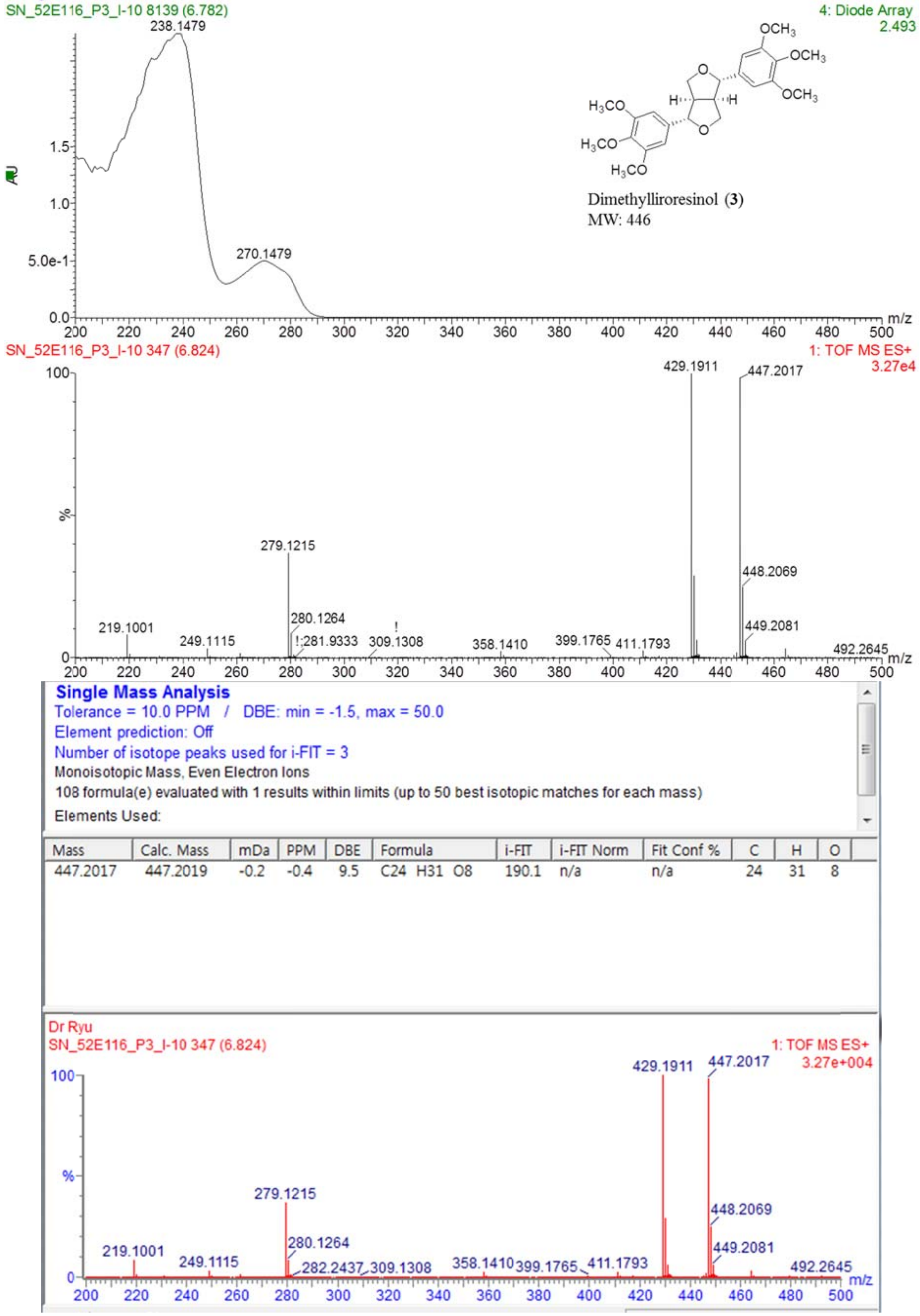

Mass	Calc. Mass	mDa	PPM	DBE	Formula	i-FIT	i-FIT Norm	Fit Conf %	C	H	O
447.2017	447.2019	-0.2	-0.4	9.5	C24 H31 O8	190.1	n/a	n/a	24	31	8

Figure S2E. UV, MS/MS, MS and HREIMS data of lignan **3**.

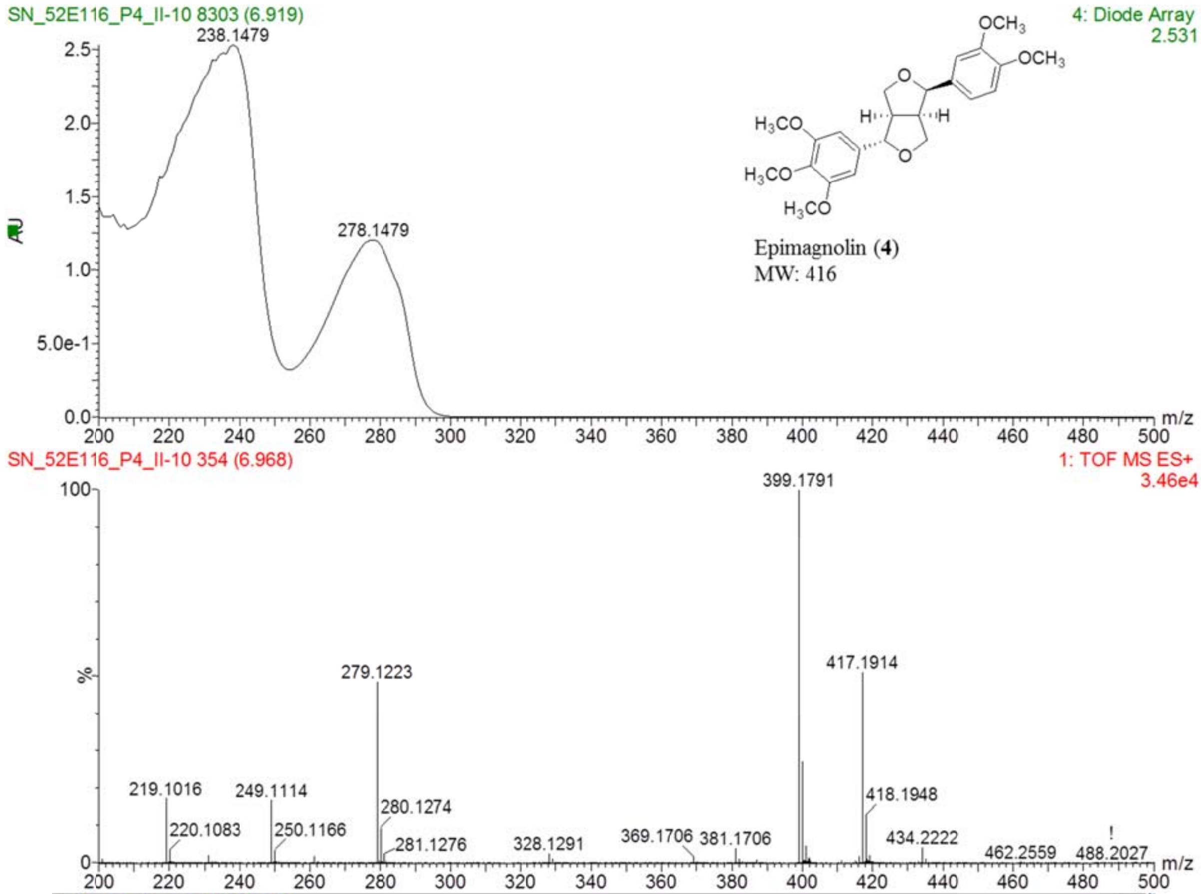

Single Mass Analysis
Tolerance = 10.0 PPM / DBE: min = -1.5, max = 50.0
Element prediction: Off
Number of isotope peaks used for i-FIT = 3
Monoisotopic Mass, Even Electron Ions
93 formula(e) evaluated with 1 results within limits (up to 50 best isotopic matches for each mass)
Elements Used:

Mass	Calc. Mass	mDa	PPM	DBE	Formula	i-FIT	i-FIT Norm	Fit Conf %	C	H	O
417.1914	417.1913	0.1	0.2	9.5	C23 H29 O7	202.8	n/a	n/a	23	29	7

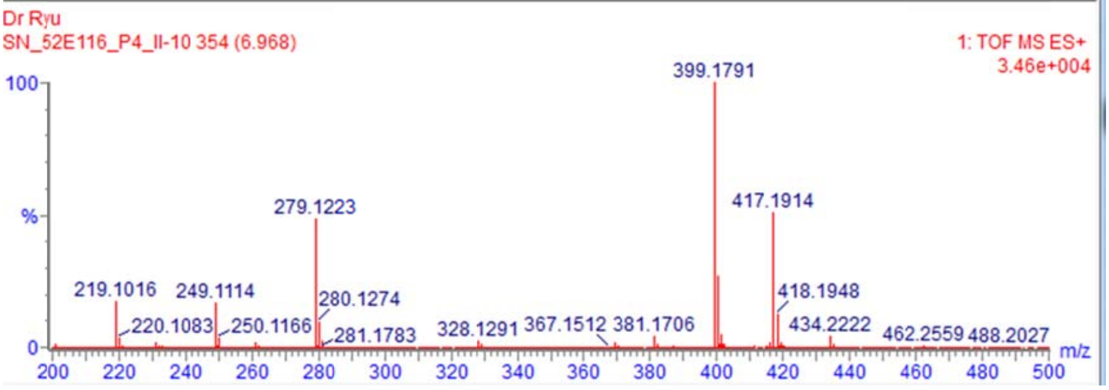

Figure S2F. UV, MS/MS, MS and HREIMS data of lignan **4**.

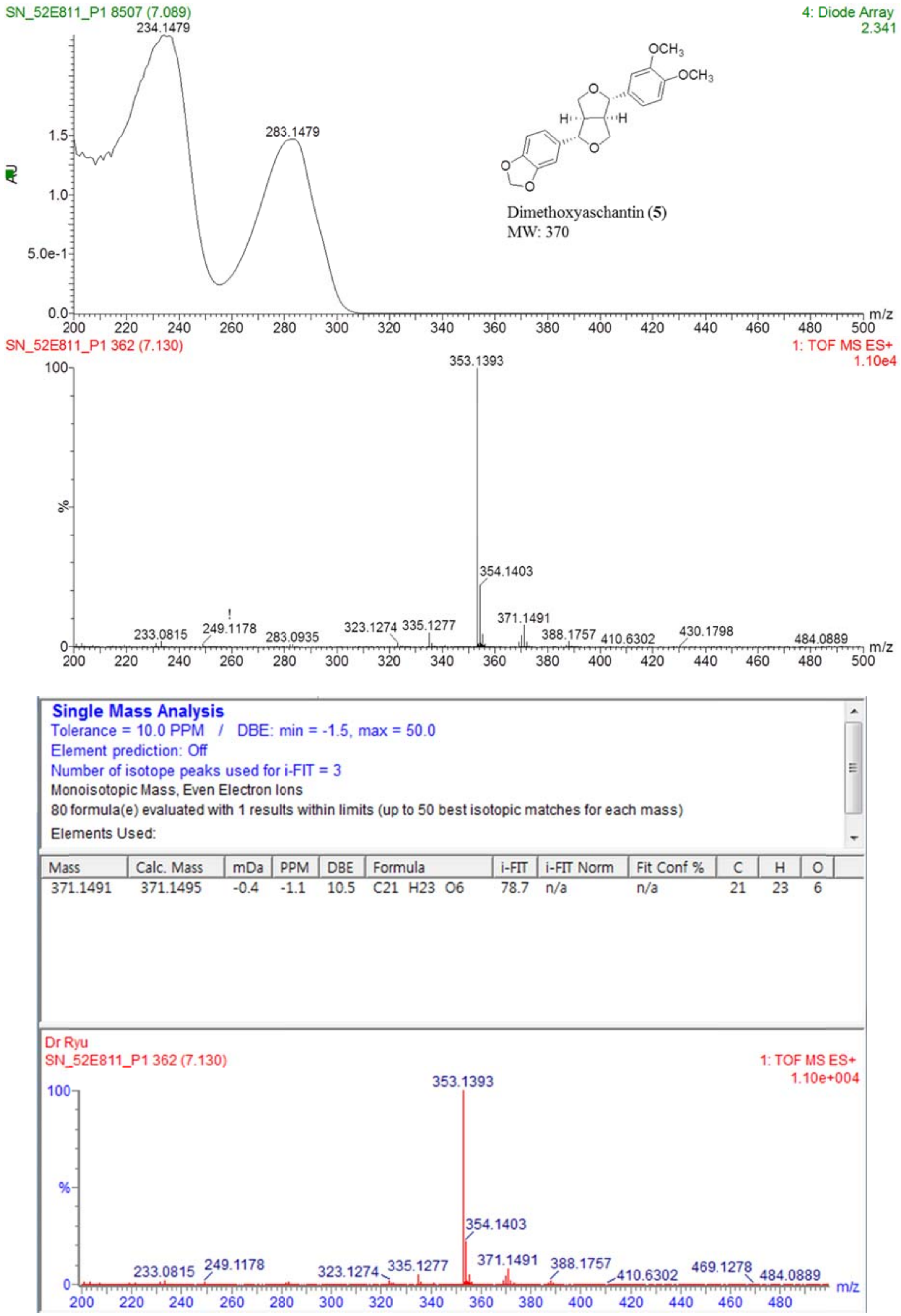

Mass	Calc. Mass	mDa	PPM	DBE	Formula	i-FIT	i-FIT Norm	Fit Conf %	C	H	O
371.1491	371.1495	-0.4	-1.1	10.5	C21 H23 O6	78.7	n/a	n/a	21	23	6

Figure S2G. UV, MS/MS, MS and HREIMS data of lignan **5**.

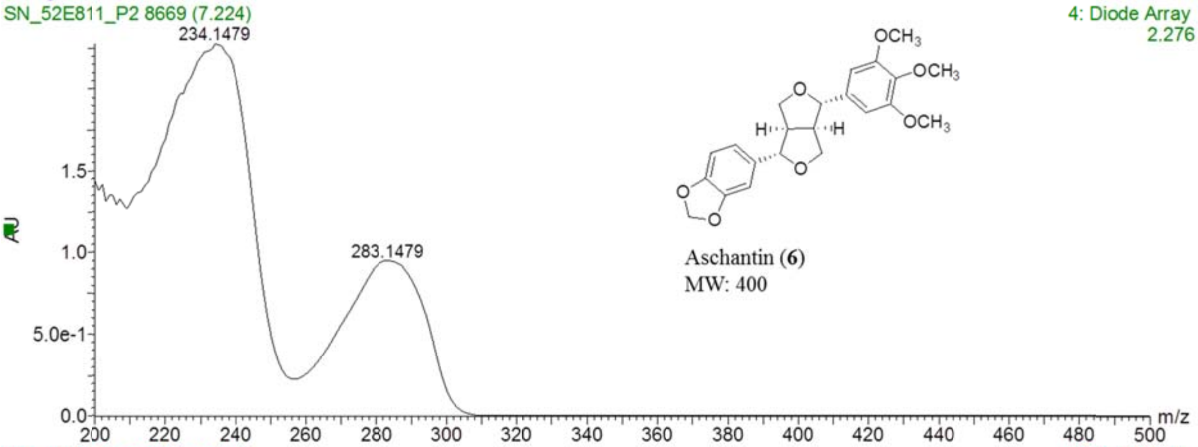

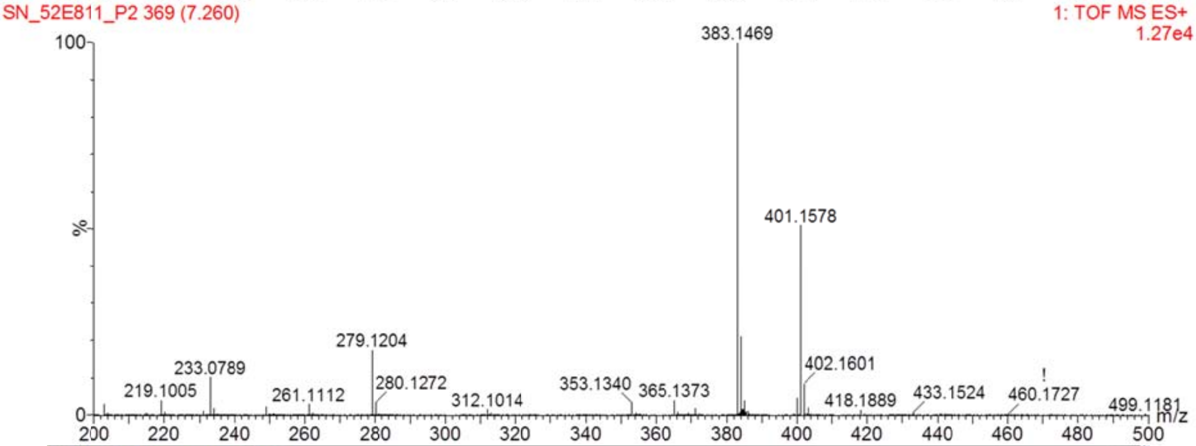

Single Mass Analysis

Tolerance = 10.0 PPM / DBE: min = -1.5, max = 50.0

Element prediction: Off

Number of isotope peaks used for i-FIT = 3

Monoisotopic Mass, Even Electron Ions

87 formula(e) evaluated with 2 results within limits (up to 50 best isotopic matches for each mass)

Elements Used:

Mass	Calc. Mass	mDa	PPM	DBE	Formula	i-FIT	i-FIT Norm	Fit Conf %	C	H	O
401.1578	401.1600	-2.2	-5.5	10.5	C22 H25 O7	89.2	0.093	91.08	22	25	7
	401.1542	3.6	9.0	19.5	C29 H21 O2	91.5	2.417	8.92	29	21	2

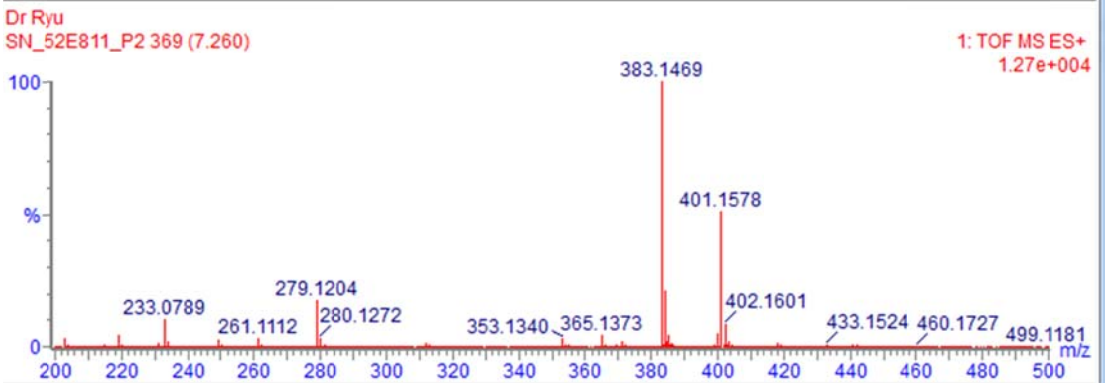

Figure S2H. UV, MS/MS, MS and HREIMS data of lignan **6**.

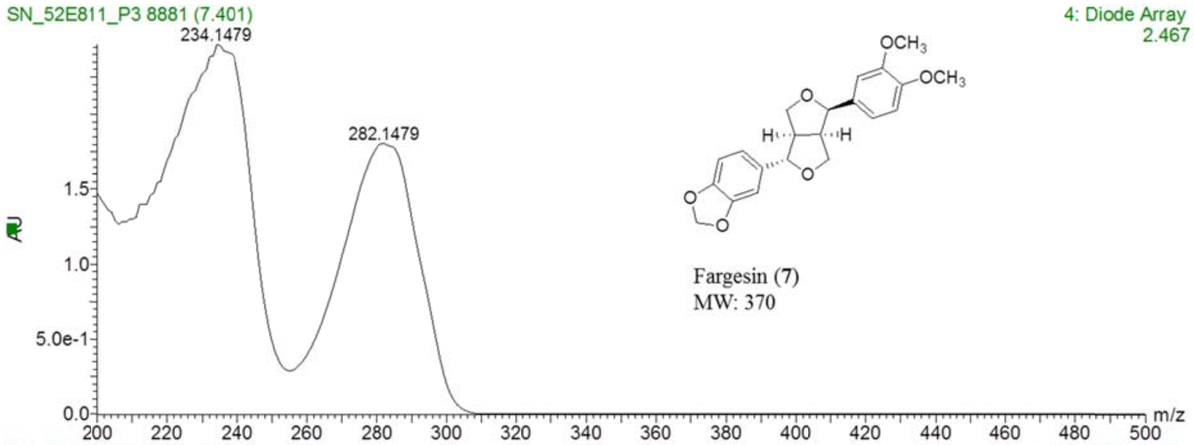

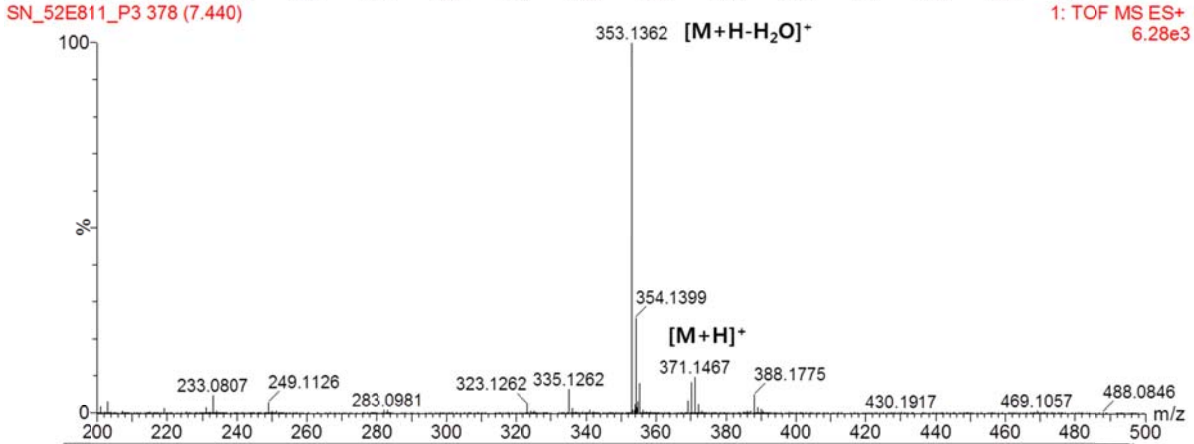

Single Mass Analysis
Tolerance = 10.0 PPM / DBE: min = -1.5, max = 50.0
Element prediction: Off
Number of isotope peaks used for i-FIT = 3
Monoisotopic Mass, Even Electron Ions
80 formula(e) evaluated with 2 results within limits (up to 50 best isotopic matches for each mass)
Elements Used:

Mass	Calc. Mass	mDa	PPM	DBE	Formula	i-FIT	i-FIT Norm	Fit Conf %	C	H	O
371.1467	371.1495	-2.8	-7.5	10.5	C21 H23 O6	79.4	0.590	55.45	21	23	6
	371.1436	3.1	8.4	19.5	C28 H19 O	79.6	0.809	44.55	28	19	1

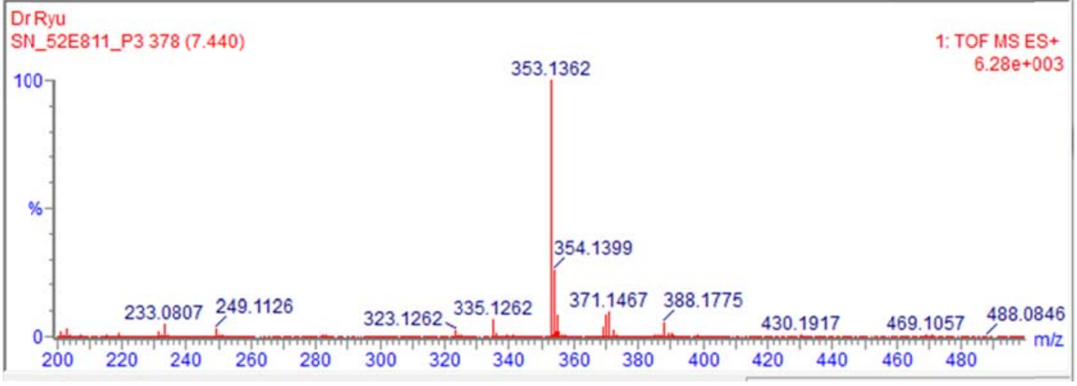

Figure S2I. UV, MS/MS, MS and HREIMS data of lignan **7**.

Figure S3

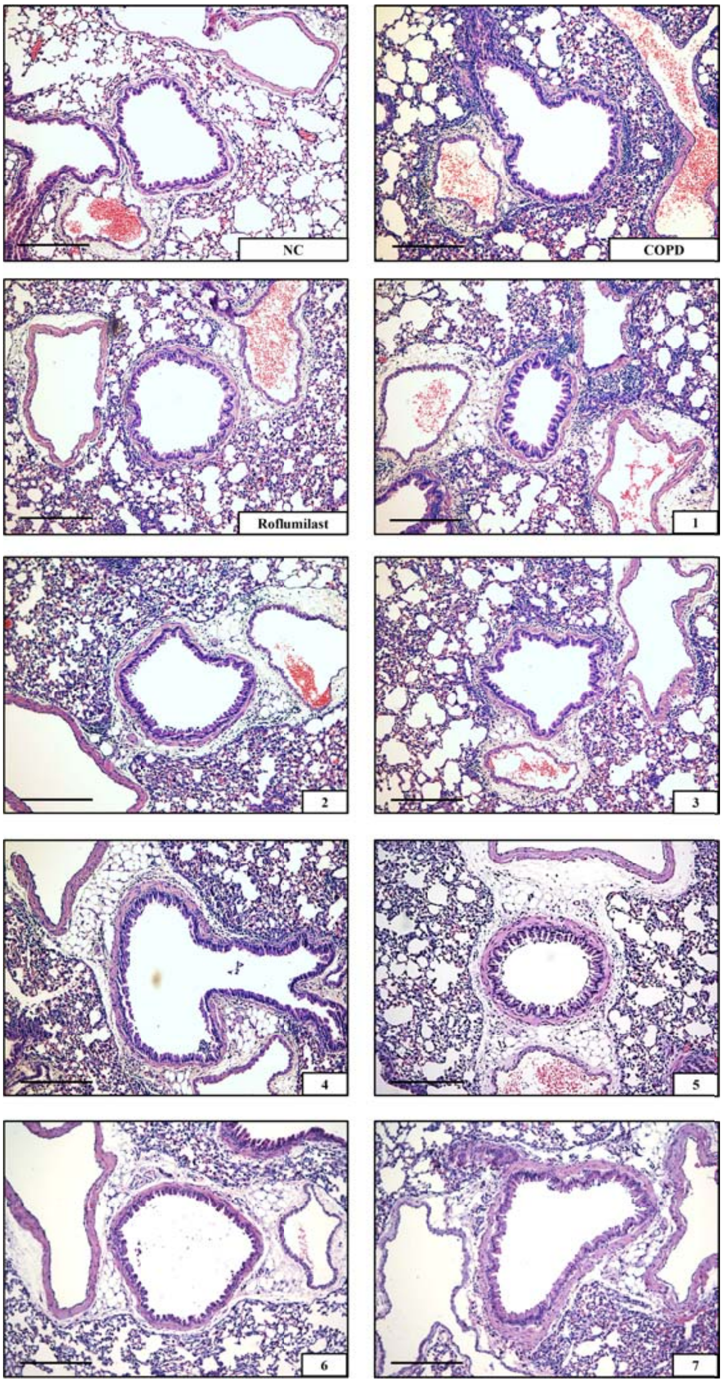

Figure S3. Airway inflammation in lung tissue of mice exposed to CS and LPS was ameliorated by the seven lignans (**1**-**7**). Representative figures for H&E staining of lung tissue. NC: normal control mice; COPD: CS/LPS exposed mice; Roflumilast: CS and LPS exposed mice treated with roflumilast (10 mg/kg); 1 to **7**; CS/LPS exposed mice treated with each of the seven lignans (15 mg/kg). Scales bars, 200 μm.

Figure S4

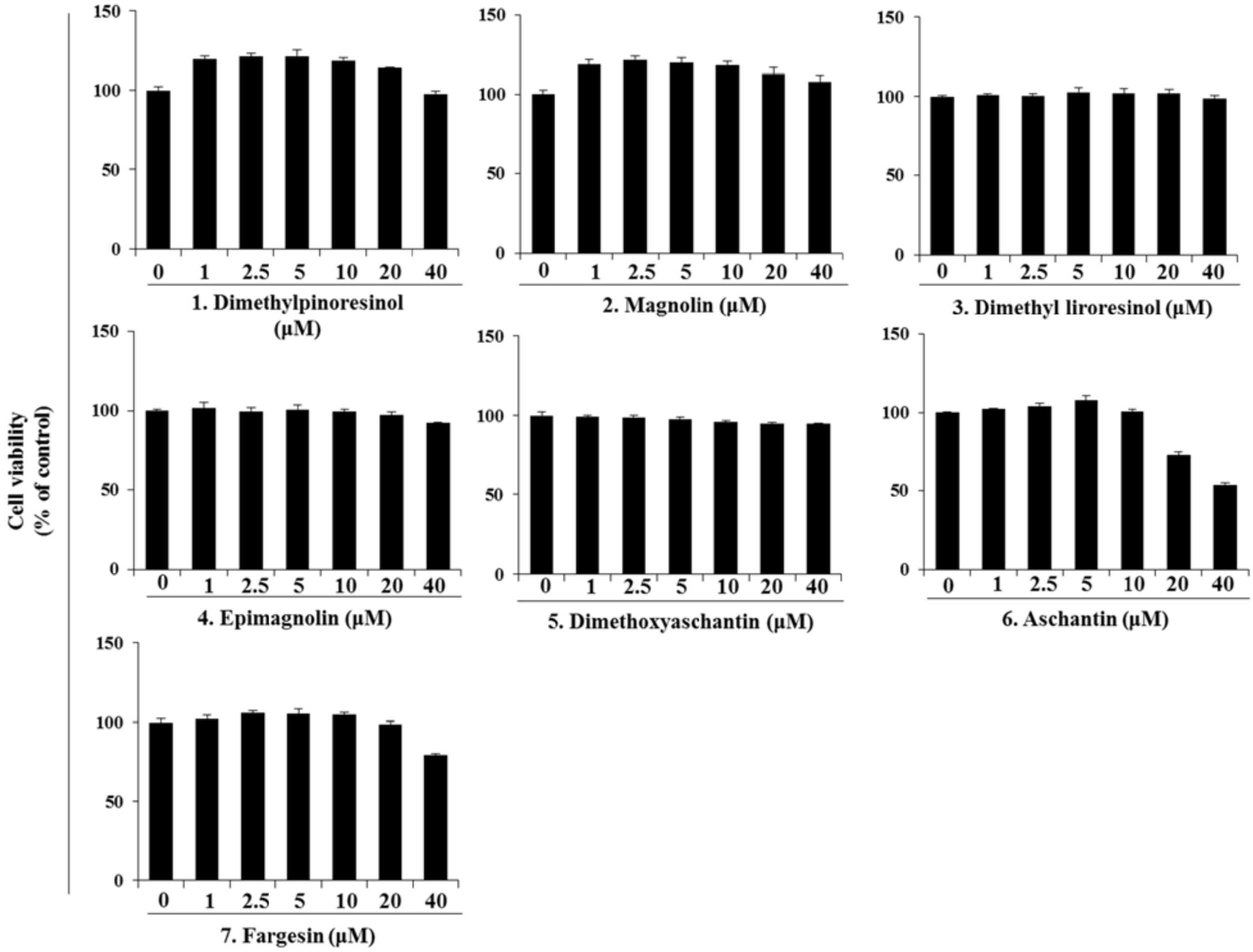

Figure S4. The seven lignans isolated from Xinyi have no cytotoxicity at concentrations below 10 μM.

Figure S5.

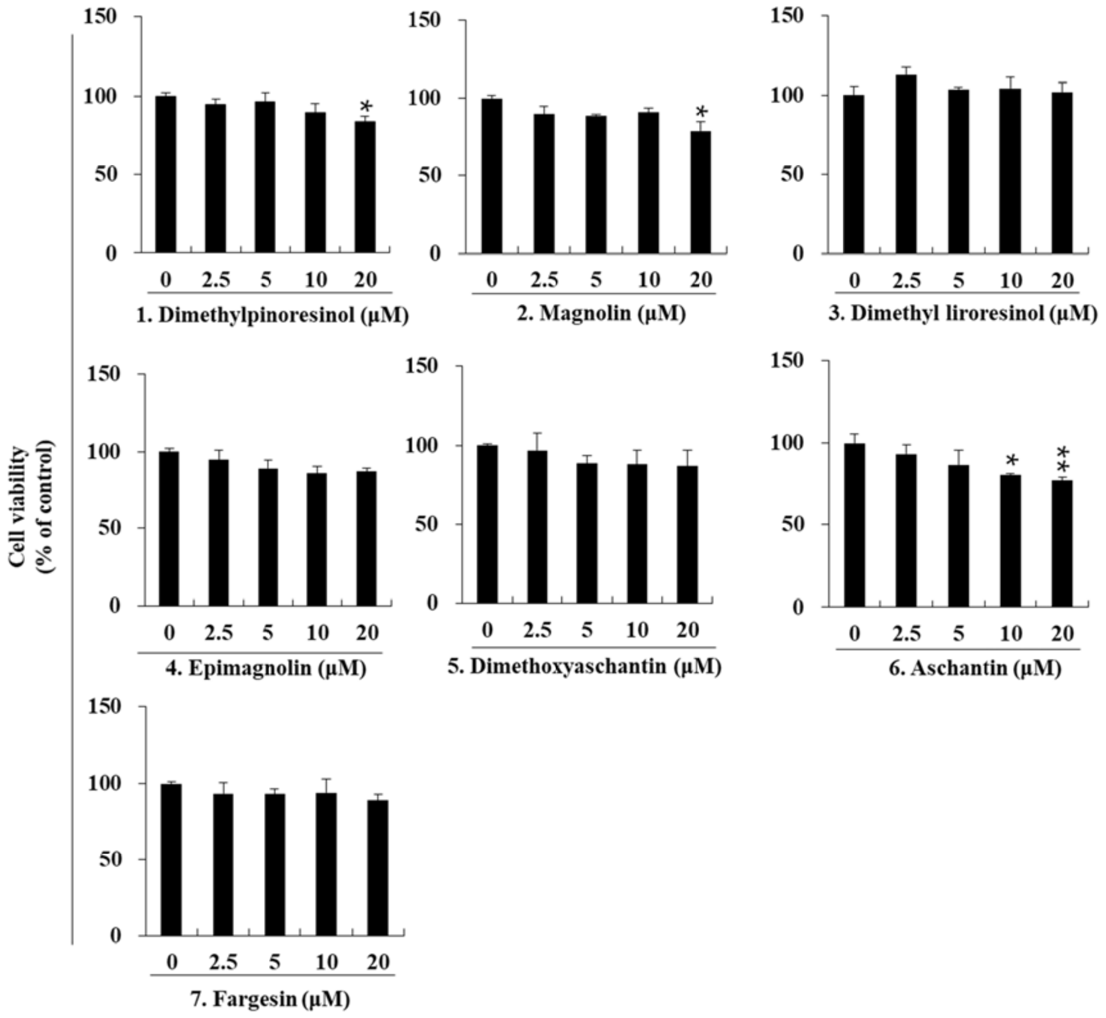

Figure S5. The seven lignans isolated from Xinyi at concentrations below 10 μM have no cytotoxicity in a condition of high cell proliferation, except for lignan **6**. The cells were cultured with the lignans in RPMI containing both 10% FBS and each concentration of lignans for 24 h. In highly proliferating cells, only lignan **6** showed weak cytotoxicity at 10 μM concentration. Lignan **1**, **2**, and **6** exhibited weak cytotoxicity at 20 μM.

Methods for Supplementary Table S1

Protein structure and compound preparation for computational calculations

We prepared five protein kinases, ERK-1, ERK-2, MEK-1, MEK-2, and AKT-1 whose three-dimensional structures are available in the Protein Data Bank (PDB), 4QTBA, 4QTEA, 3DV3A, 1S9IA, 3O96A, and 1O6LA, respectively. Because these structures have missing residues, which were not revealed by experimental methods, we prepared these residues using CHARMM program (Chemistry at HARvard Macromolecular Mechanics: http://www.charmm.org). The ideal topology information for these absent residues was used to generate their missing atoms with the CHARMM commands, ic para and ic build. Three dimensional coordinates of the seven compounds were prepared using the Marvin program (ChemAxon; http://www.chemaxon.com; 5.11.4, 2012). Prior to performing protein-ligand docking simulations, five kinases and seven compounds were prepared.

Protein-compound docking simulations

Protein-compound docking simulations were conducted using the AutoDock Vina program (http://vina.scripps.edu). A protein structure has its own pockets that can interact or bind with a ligand. These pockets were searched using Pck pocket detection software (http://schwarz.benjamin.free.fr/Work/Pck/home.htm). Of these pockets, we chose those with pocket volumes greater than 100 $Å^3$. Each pocket consisted of protein residues. The identified residues act as a starting coordinate for protein-compound docking simulations. Ten docking simulations with a different random seed for each residue were performed. To prevent the drifting of a ligand from a starting coordinate, a virtual box with a length of 15 Å was used. Thousands of compound docking doses were calculated, which were grouped based on their three dimensional coordinates. We chose the first- and second-lowest conformations in the groups and visually examined where they were bound. These results are tabulated in **Table S1**.

Table S1. Molecular docking study of MEK1/2, ERK1/2 or AKT1 with seven lignans (**1**-**7**) (energy: kcal/mol and docking positions[a]).

	Compound	MEK-1	MEK-2	ERK-1	ERK-2	AKT-1
**1**	dimethyl_pinoresinol	-8.3 ATP<br>-7.2 near	-7.7 ATP<br>-7.6 near	-7.4 near<br>-7.4 ATP	-7.7 ATP<br>-7.0 Surface	**-9.3** ATP<br>**-9.0** ATP
**2**	magnolin	-7.7 ATP<br>-7.0 near	-7.6 near<br>-7.4 ATP	-7.4 near<br>-7.4 ATP	-7.5 ATP[b]<br>-7.1 ATP[b]	-8.9 ATP<br>-8.8 ATP
**3**	dimethyl_liroresinol	-7.1 near<br>-6.7 ATP	-7.4 near<br>-7.3 ATP	-7.7 near<br>-7.3 ATP	-7.6 ATP<br>-6.6 near	**-9.0** ATP<br>-8.4 near
**4**	epimagnolin	-7.8 near<br>-7.4 near	-7.6 near<br>-7.4 ATP	-7.6 near<br>-7.6 ATP	-7.8 ATP<br>-6.9 Surface	-8.9 near<br>-8.7 ATP
**5**	demethoxyaschantin	-8.5 near<br>-7.7 near	-8.4 near<br>-8.3 near	-8.5 near<br>-8.1 ATP	**-9.1** ATP<br>-7.8 Surface	**-9.9** ATP<br>**-9.2** ATP
**6**	aschantin	-8.3 near<br>-7.5 near	-8.1 near<br>-8.0 near	-8.0 ATP<br>-7.7 Surface	-8.7 ATP<br>-7.8 ATP	**-9.7** ATP<br>**-9.1** near
**7**	fargasin	-8.5 near<br>-7.7 near	-8.4 near<br>-8.3 near	-8.7 near<br>-8.2 ATP	**-9.2** ATP<br>-8.2 near	**-9.9** ATP<br>**-9.2** near

[a]Docking positions:
ATP: a chemical on the ATP binding site
Near: a chemical near the ATP binding site (it could be on the entrance)
Surface: a chemical on a surface exterior to the ATP binding site
[b]the first and second-lowest energy chemicals can exist in the same ATP pocket because the ATP pocket is large.
